# Integrative analysis of *SoARF* gene family uncovers their role in hormone signaling and development in sugarcane

**DOI:** 10.3389/fpls.2026.1874213

**Published:** 2026-06-26

**Authors:** Bao Wen-Qing, Zhang Ming-Hui, Liang Cui-Li, Li He-Ping, Ahmad Ali, Li Rui-Mei, Zhang Yue-Bin, Zhang Shu-He

**Affiliations:** 1 Institute of Subtropical Agriculture, Fujian Academy of Agriculture Sciences, Zhangzhou, China; 2 State Key Laboratory of Tropical Crop Breeding, Key Laboratory of Biology and Genetics Resources of Tropical Crops Ministry of Agriculture and Rural Affairs, Key Laboratory of Conservation and Utilization of Tropical Agricultural Biological Resources of Hainan Province, Institute of Tropical Bioscience and Biotechnology, Chinese Academy of Tropical Agricultural Sciences, Haikou/Sanya, Hainan, China; 3 State Key Laboratory of Tropical Crop Breeding, Sugarcane Research Institute, Yunnan Academy of Agricultural Sciences, Kaiyuan, China

**Keywords:** ARF TFs, auxin signaling, genome-wide analysis, *S. officinarum*, sugarcane development

## Abstract

Auxin Response Factors (ARFs) are key regulators of auxin-based transcriptional networks regulating plant development and stress responses. Nevertheless, their systematic description in cultivated sugarcane (*Saccharum officinarum*) is still not very well described since it has a complicated polyploid genome. A genome-wide analysis in this study revealed that 73 *ARF* genes (*SoARFs*) were detected in the *S. officinarum* LA-Purple genome, indicating substantial expansion in gene families in comparison to the diploid species. Differences in protein length, molecular weight, and isoelectric point indicate structural differences and possible functional divergence. Phylogenetic analysis types *SoARFs* into two major clades (II and IV), with no other clades being present, suggesting the lineage-specific evolutionary patterns, and all conserved relations with monocot *ARFs*, suggesting evolutionary conservation. Conserved domains (B3, Auxin resp, AUX/IAA) were confirmed by structural analysis, as well as variable exon-intron organization, indicating regulatory diversification. The analysis of chromosomal distributions and synteny showed that the prevalent way of expanding the number of genes in the family was due to segmental and whole-genome duplication, demonstrating polyploidy-based evolution. Promoter analysis revealed that 49 *cis*-regulatory elements linked to phytohormone signaling and stress responses, which means that *SoARFs* are integrative regulators of developmental and environmental pathways. Further regulatory network analysis identified multilayered regulation with widespread miRNA targeting especially of miR160 and miR167, and multifaceted protein-protein interactions. The spatial and temporal dynamics of expression profiling in exogenous IAA conditions and different stages of development indicated unique patterns with major genes (*SoARF1*, *SoARF2*, *SoARF3*, *SoARF6, SoARF7*, *SoARF12*, and *SoARF19*) being highly induced and tissue-specific. Furthermore, qRT-PCR confirmed that *SoARF43, SoARF44, SoARF48*, and *SoARF71* were identified as negative regulator of IAA. These results demonstrate the functional specialization in the ARF family and lay a basis on future functional research and genetic enhancement of sugarcane.

## 1 Introduction

Sugarcane is one of the most important industrial crops worldwide and remains the primary source of sucrose as well as a major feedstock for bioenergy and bioproducts. Despite its economic value, the genetic dissection of key regulatory pathways in sugarcane has lagged behind that of many diploid crops because of its large, polyploid, and highly complex genome (Healey et al., 2024). The current sugarcane cultivars (*Saccharum* spp. hybrid, 2n = 100-130) are highly polyploid and aneuploid interspecific hybrids between *S. officinarum* (2n = 8x = 80) and *Saccharum spontaneum* (2n = 4x-16x = 40-128) (Zhang et al., 2022; Kumar et al., 2023). This challenge is especially relevant in *S. officinarum*, the noble cane species that contributes substantially to cane quality and biomass-related traits in modern breeding programs. Subsequently, the availability of genomic resources now creates an opportunity to move from descriptive phenotypes to regulatory mechanisms, particularly for transcription factor (TF) families that coordinate growth, development, and environmental responsiveness in cultivated sugarcane.

The genome of modern sugarcane cultivars is complex, with high levels of polyploidy and aneuploidy (2n = 100-130). Of this complexity, 80% originates from *S. officinarum* (2n = 80) and 10-15% from *S. spontaneum* (2n = 40-128). Recent sugarcane genome resources have strengthened gene family analysis in *Saccharum* species. The *S. spontaneum* AP85–441 and Np-X genomes clarified autopolyploid genome organization, chromosome reduction, and polyploid evolution, while chromosome-scale assemblies of modern sugarcane hybrids such as R570 and ZZ1 revealed extensive haplotype redundancy, interspecific recombination, homeolog retention, and subgenome-specific gene expansion (Zhang et al., 2018, 2022; Healey et al., 2024; Bao et al., 2024). Together with *Saccharum*-focused databases such as *ScDB*, these resources allow TF families to be investigated within a sugarcane-specific polyploid and syntenic framework rather than by relying mainly on diploid model crops (Chen et al., 2024).Among plant hormonal pathways, auxin is distinguished by the breadth of processes, it regulates from embryonic patterning and organ initiation to vascular differentiation, tropic growth, and reproductive development (Gomes and Scortecci, 2021). In the canonical nuclear auxin pathway, *Auxin Response Factors (ARFs)* serve as the principal transcriptional output downstream of TIR1/AFB receptors and Aux/IAA repressors (Li et al., 2023). Structurally, *ARFs* typically comprise an N-terminal B3-type DNA binding domain (DBD), a middle region that determines transcriptional activation or repression, and a carboxyl (C)-terminal PB1 containing dimerization domain (CTD) that mediates ARF-ARF and ARF-Aux/IAA interactions (Li et al., 2025; Lin et al., 2023). Foundational studies established that *ARFs* bind AuxREs (TGTCNN) containing promoters of the targeted genes to activate or suppress expression, while variations in domain composition and DNA binding specificity generate diverse transcriptional outputs from auxin signaling (Rienstra et al., 2025).

Genome-wide analyses across plant lineages demonstrate that the *ARF* gene family is conserved in core architecture but highly variable in size and composition, ranging from only a few members in lower plants to large multigene families in higher plants (Rienstra et al., 2025). *ARF* repertories have been shown to expand through duplication events and subsequently diversify gene structure motif composition and regulatory elements. Sugarcane-specific studies of auxin-signaling gene families, including GH3 and Aux/IAA, show that polyploidization, segmental duplication, and recombination contributed to gene-family expansion and tissue-, stress-, and regeneration-related expression divergence in *Saccharum*, supporting the need for *ARF* characterization in cultivated *S. officinarum* (Zou et al., 2022; Huang et al., 2024). For instance, extensive variation in *ARF* family size has been reported across taxa, including large expansions in orchid species and polyploid crops such as wheat (Feng et al., 2024; Chandra et al., 2020). These reflect in differences in *cis* regulatory elements, tissue specific expression, and hormone reaction. In crop systems, such integrative analyses combining phylogeny, chromosomal distribution, conserved domains, and expression profiling have provided key insights into *ARF* evolution and function. Beyond their roles in development, *ARFs* are increasingly recognized as central regulators linking auxin signaling with environmental adaptation and stress responses (Wilkinson et al., 2026). Numerous studies have demonstrated that *ARF* genes respond under biotic and abiotic stresses, especially the pathogen infection, salinity, drought, and temperature fluctuations (Cao et al., 2025; Liu et al., 2024). For example, differential expression of *ARF* genes has been observed under pathogen challenge in *Solanum lycopersicum* and *Triticum aestivum* L., while specific *ARFs* such as *OsARF17* play critical role in antiviral defense in *Oryza sativa* L (Song et al., 2023; Lin et al., 2023). Similarly, *ARF*-mediated regulatory networks have been implicated in responses to salicylic acid, abscisic acid, and osmotic stress across multiple species (Das et al., 2026). These findings highlight the functional plasticity of *ARFs* and their involvement in integrating hormonal signaling with environmental cues. In addition to stress adaptation, *ARFs* also participate in specialized metabolic regulation. Functional studies in *Malus domestica* have demonstrated that *MdARF2* negatively regulates anthocyanin accumulation, indicate that *ARFs* extend their influence beyond classical developmental processes to secondary metabolism (Wang et al., 2025).

Transactivation and transient overexpression experiments indicated that *RrMDHAR1* is activated by *RrARF5*, indicating that *ARF* genes are involved in the regulation of vitamin C accumulation in *Rosa roxburgii* (Feng et al., 2025). In sugarcane, genome-wide identification of *ARFs* in *S. spontaneum* revealed conserved domain organization, uneven chromosomal distribution, and expansion driven by segmental duplication, along with differential expression under pathogen infection, and hormonal treatment (Huang et al., 2024). These findings suggest that sugarcane *ARFs* exhibit both evolutionary conversion and functional divergence. Despite these advances, the *ARF* gene family remains insufficiently characterized in cultivated *S. officinarum*. Studies based on wild or related *saccharum* genome provide valuable references but do not fully resolve the genomic organization, duplication events, regulatory architecture, and expression dynamics of *ARFs* in noble cane.

Given the distinct genomic background and agronomic importance of *S. officinarum* LA-Purple, a comprehensive analysis is required to elucidate the expression, diversification, and functional potential of *ARF* family members in this genotype. Given the extensive expansion of TF families in polyploid sugarcane genomes, it remains unclear whether *ARF* gene-family diversification contributes to auxin-mediated developmental regulation in cultivated *S. officinarum*. Therefore, this study was designed to address two key questions: (i) how polyploidization and duplication events have shaped the evolution and diversification of the *SoARF* gene family, and (ii) which *SoARF* members are potential regulators of auxin-mediated developmental processes based on their regulatory architecture and expression patterns. By integrating phylogenetic, structural, promoter, miRNA, protein interaction, tissue-specific expression, and IAA-responsive expression analyses, we aimed to identify candidate *SoARF* genes associated with sugarcane growth and development. This study provides a systematic framework for understanding the evolutionary expansion and biological roles of *ARF* genes in Nobel cane.

## 2 Materials and methods

### 2.1 Sequence retrieval of ARF gene family from *S. officinarum* LA-Purple genome

The genome sequences of *S. officinarum* LA-Purple were downloaded from the Sugarcane Genome Database (ScBD: https://sugarcane.gxu.edu.cn/). To identify *saccharum* ARF family genes, a BLASTp search was performed using 35 *ZmARF* gene sequences from maize (Liu et al., 2011), obtained from the maize genome database, as queries (score threshold ≥ 100, e-value ≤ 1 × 10^-5^). Additionally, the HMMER 3.0 tool was employed to search against hidden Markov model (HMM) profiles corresponding to the ARF domains PF06507, PF02309, and PF02362 (e-value ≤ 0.001). Candidate genes were further validated by domain annotation using InterProScan (https://www.ebi.ac.uk/interpro/about/interproscan/) and online Pfam database (http://xfam.org/). Sequences with no valid ARF family members domain and partial domain were excluded and removed from analysis manually. Finally, a total of 73 *SoARF* genes were identified in the *S. officinarum* LA-Purple genome.

### 2.2 Prediction of physiochemical properties and orthologous gene clusters

The physicochemical properties, including molecular weight (Mw) and theoretical isoelectric point (pI), of *SoARF* proteins were analyzed with the ProtParam tool on the Expasy website (https://web.expasy.org/) according to (Rehman et al., 2026). The subcellular localization of the *SoARF* proteins was predicted by the WoLF PSORT server (https://wolfpsort.hgc.jp/). The amino acids length (aa), coding sequence (CDS) length (bp), gene length (bp), instability index (II), and GRAVY were predicted using Batch Protein Parameter Calc module in TBtools-II software (v2.454) (Chen C. et al., 2023). The identified ARF proteins sequences from *S. spontaneum* AP85-441, *S. officinarum* LA-Purple, *S. spontaneum* Np-X, and *S. bicolor* were used for Orthologous gene clustering using OrthoVenn3 online comparative genomics web tool (https://orthovenn3.bioinfotoolkits.net/home) based on whole-genome and ARF family protein sequences of these species.

### 2.3 Multiple sequence alignments and phylogenetic analysis

Multiple sequence alignments were carried out using MAFFT (v7.407) employing default parameters. The resulting alignment file was then subjected to ESPript (v3.2, https://espript.ibcp.fr) for sequence visualization and figure generation. The protein sequences of *SoARF* genes were subsequently extracted from the genome datasets of the *S. officinarum* LA-Purple. Multiple sequence alignments of SoARF protein sequences were performed. For the comparative phylogenetic analysis, the ARF protein sequences were retrieved from the available public genome databases using *S. spontaneum* AP85-441 (https://sugarcane.gxu.edu.cn), *O. sativa* (release 20), *S. bicolor* (release 29), *Zea mays* (release 29), and *Triticum aestivum* (release 62) from Ensembl Genomes online database (https://ensemblgenomes.org). The best-fit substitution model was determined using IQ-TREE2 based on the lowest Bayesian Information Criterion (BIC) value. A maximum likelihood (ML) phylogenetic tree was then constructed using IQ-TREE2 under the best-fit model, with bootstrap support calculated from 1,000 replicates (Minh et al., 2020). The resulting tree was visualized using online iTOL server (https://itol.embl.de/).

### 2.4 Prediction of gene structure, conserved domain and conserved motif

The gene structure (exon-intron distribution) of the identified *SoARF* genes was analyzed using the “Gene Structure View” module in TBtools (v2.454) based on their coding sequences (CDS) and corresponding protein sequences. The deduced SoARF protein sequences were further validated by functional domain prediction with the CDD-batch (Conserved Domain Database) tool available from NCBI (https://www.ncbi.nlm.nih.gov). Conserved motifs within these SoARF proteins were characterized using the online MEME Suite (v5.0.2) (https://meme-suite.org/meme/), with the search set to identify up to 11 motifs in the input sequences (Saleem et al., 2022). Motif distribution was subsequently visualized using the “Motif Pattern” program in TBtools (v2.454) (Chen C. et al., 2023).

### 2.5 *Cis*-regulatory analysis

Promoter sequences, defined as the 1,500 bp region upstream of the start codon (ATG) for all *SoARF* genes, were retrieved from the corresponding genome assemblies. Putative cis-regulatory elements within these promoter sequences were subsequently identified and annotated using the PlantCARE online database (https://bioinformatics.psb.ugent.be/webtools/plantcare/html/) and visualized through “Simple BioSequence Viewer” module in TBtools (v2.454) (Chen C. et al., 2023).

### 2.6 Chromosomal location, gene duplication, and synteny analysis

The genomic location information of each *SoARF* gene was extracted from the GFF3 genome annotation files of *S. officinarum* LA-Purple and visualized using the “Gene Location Visualize from GTF/GFF” module in TBtools (v2.454) (Chen C. et al., 2023). For gene duplication and synteny analysis, whole-genome protein sequences of the target species and reference species were combined and subjected to all-to-all BLAST (DIAMOND) with an E-value threshold of 1e^-10^. The resulting alignments were filtered to retain only those with a minimum sequence identity of 90% and a minimum coverage of 90%, where coverage was defined as the maximum ratio of alignment length to the length of the query or subject protein (Lin et al., 2023). Subsequently, syntenic blocks and duplicated genes were identified using MCScanX (Super-fast) toolkit with default parameters, and the syntenic regions were visualized with the “Multiple Synteny Plot” module TBtools (v2.454) (Chen C. et al., 2023).

### 2.7 Protein-protein interaction analysis and prediction of miRNAs targeting *SoARF*

PPI interactions among SoARF proteins from *S. officinarum* LA-Purple and their orthologs in *S. bicolor* were predicted using the STRING database (v12.0, https://string-db.org) with default parameters (Saleem et al., 2022). Putative miRNAs targeting *SoARF* genes in *S. officinarum* LA-Purple were predicted using the psRNATarget server (https://www.zhaolab.org/psRNATarget/). The coding sequences (CDS) of *S. officinarum* ARF genes were used as target sequences, while known miRNA sequences from *Z. mays* were selected as the reference library, given the evolutionary proximity between the two-grass species. The analysis was conducted under default parameters, and only high-confidence predictions were retained for further analysis. A schematic diagram depicting the interaction networks between miRNAs and their targeted *S. officinarum* LA-Purple ARF genes was generated using Cytoscape (v3.10.4) (https://cytoscape.org/).

### 2.8 Expression analysis of *SoARF* genes

Expression profiles of the 73 identified *SoARF* genes were analyzed using publicly available transcriptome datasets retrieved from the Sugarcane Genome Database (ScDB: https://sugarcane.gxu.edu.cn/). Two types of expression datasets were used: tissue/developmental-stage expression data and exogenous IAA-treatment expression data. For the developmental-stage analysis, samples represented different growth stages and tissues of sugarcane, including leaf and stem tissues collected at the seedling stage, defined as 35 days after planting/sowing. In addition, tissues from the premature stage at 9 months and the mature stage at 12 months were included. These samples consisted of rolled leaf, fully expanded leaf, and stem tissues at different developmental positions. Stem3, stem9, and stem15 refer to the third, ninth, and fifteenth stem nodes/internodes counted from the shoot apex, respectively. Stem3 represents the upper immature stem region, stem9 represents the middle premature/intermediate stem region, and stem15 represents the lower mature basal stem region.

For the hormone-response analysis, transcriptome data from seedling-stage leaf and stem tissues exposed to exogenous indole-3-acetic acid treatment were used. Samples were collected at 24, 48, and 96 h after IAA treatment, and corresponding untreated samples collected at the same developmental stage were used as controls. Gene expression values were quantified as FPKM, and log2-transformed fold-change values were used to visualize expression patterns. Genes with |log2 fold change| > 1.0 and *p <* 0.05 were considered significantly differentially expressed. Heatmaps were generated using the ComplexHeatmap package in R.

#### 2.8.1 IAA treatment and sample collection

For RT-qPCR validation, sugarcane seedlings of the widely cultivated cultivar GT58 were employed. Derived from the cross of YT85-177 × CP81-1254, GT58 is noted for its high and stable cane yield, medium maturity, elevated sugar content, robust resistance to smut disease, and moderate drought tolerance. Plants were grown under uniform conditions until the early seedling stage, corresponding to 35 days after planting. This cultivar was used for RT-qPCR validation only, while the RNA-seq expression analysis was based on publicly available *S. officinarum* LA-Purple transcriptome data. Seedlings at the same developmental stage and with similar growth status were selected for hormone treatment. The IAA treatment was performed by spraying plants with 100 μM indole-3-acetic acid (IAA) solution until runoff, while control plants were sprayed with distilled water under the same conditions. Leaf and stem tissues were collected from both IAA-treated and control plants at 24, 48, and 96 h after treatment. For each treatment and time point, three independent biological replicates were collected, and each replicate consisted of tissues pooled from comparable plants to minimize individual variation. All samples were immediately frozen in liquid nitrogen and stored at -80 °C until RNA extraction.

#### 2.8.2 Total RNA extraction and cDNA synthesis

Total RNA was extracted from sugarcane leaves and stem using the TRIzol reagent according to the manufacturer’s instructions. RNA purity and integrity were assessed by measuring OD values and by electrophoresis on a 1% agarose gel. First-strand cDNA was synthesized from total RNA using the HiScript II Q RT SuperMix for qPCR (+gDNA wiper) kit (Vazyme, China). The reaction mixture contained 4 μL 4× gDNA wiper Mix, 1.0 μg total RNA, and RNase-free water to a final volume of 16 μL. The mixture was incubated at 42 °C for 2 min to remove genomic DNA contamination. After cooling on ice to 4 °C, 4 μL of 5× HiScript II qRT SuperMix II was added, followed by incubation at 50 °C for 15 min and 85 °C for 5 s. The synthesized cDNA was stored at −20 °C until use.

#### 2.8.3 RT-qPCR analysis

Quantitative real-time PCR (RT-qPCR) was performed using the SYBR Green method. The qPCR reaction mixture (20 μL) consisted of 10.0 μL 2× ChamQ Universal SYBR qPCR Master Mix, 0.4 μL forward primer, 0.4 μL reverse primer, 1 μL cDNA, and 8.2 μL RNase-free water. Reactions were gently mixed, briefly centrifuged, and carried out on a QuantStudio 3 Real-Time PCR System (Applied Biosystems, USA). The amplification program was as follows: initial denaturation at 95 °C for 30 s; followed by 40 cycles of 95 °C for 10 s and 60 °C for 30 s. Specific primers for the ARF family members were designed using the PrimerQuest™ web server (https://sg.idtdna.com/pages/tools/). Details of the primers used in this study are provided in **Supplementary Table 1**. Glyceraldehyde-3-phosphate dehydrogenase (GAPDH) was used as the internal reference gene. Each sample included three biological replicates and three technical replicates. Relative gene expression levels were calculated using the 2^^−ΔΔCt^ method.

### 2.9 Statistical analysis

A Student’s *t*-test was employed to compare treated and untreated samples and assess the statistical significance of gene expression patterns. *P*-values were categorized as follows: < 0.05, ≤ 0.01, and ≤ 0.001, represented by ∗, ∗∗, and ∗∗∗, respectively. Experimental data were collected in triplicate and presented as mean ± SD. One-way and two-way ANOVA analyses were conducted using GraphPad Prism 10 for multivariate variance analysis, and bar graphs were created to illustrate the results.

## 3 Results

### 3.1 Genome-wide identification of *ARF* family genes in *S. officinarum* LA-Purple genome

For the identification of *S. officinarum* ARF, a total of 35 *ZmARF* gene sequences from the maize genome database were used as a query in a BLASTp search (score threshold ≥ 100, e-value ≤ 1 × 10^-^^5^). Furthermore, the ARF domains PF06507, PF02309, and PF02362 (e-value ≤ 0.001) were searched against Hidden Markov Model (HMM) profiles using the HMMER 3.0 program. After the domain annotation validation, finally 73 *ARF* genes were reported in the *S. officinarum* LA-Purple genome (**Table 1**). For systematic identifications, these genes were renamed based on their chromosomal positions. The protein lengths of 73 predicted SoARF members were not the same, the amino acid (aa) counts varied from 661 (SoARF33 and SoARF37) to 1155 aa in SoARF52, SoARF66, and SoARF69. This indicates that ARF proteins show substantial size variations. The predicted isoelectric point (pI) ranged from 5.54 (SoARF17) to 6.4 (SoARF60) indicating that they are slightly acidic in nature, while the molecular weights (Mw) varied from 73.2 kDa (SoARF33 and SoARF37) to 128.2 kDa in SoARF10 which suggest there is a structural diversity among the SoARF members. The Grand Average of Hydropathicity (GRAVY) values were negative indicating that they are hydrophilic in nature and showed variability from -0.384 (SoARF9) to -0.652 (SoARF3), while the instability index ranged from 54.16 (SoARF28) to 71.3 (SoARF60). Interestingly, six SoARF members (SoARF10/13/15/22/72/73) were predicted to localize in the chloroplast, only one-member SoARF60 was localized in the cytoplasm, whereas most SoARFs were predicted to be nuclear proteinswhile (**Table 1**). Since ARF proteins are generally recognized as nuclear TFs, this prediction may indicate either functional diversification of specific SoARF members or limitations of computational localization prediction. Therefore, experimental validation, such as fluorescent protein localization assays, will be necessary to confirm their actual subcellular distribution and biological significance.

**Table 1:** The basic information and physiochemical properties of *SoARF* family genes in *S. officinarum* LA-Purple genome.

S.No	Gene Name	Gene Model	Gene ID	Aaa (aa)	CDS (bp)	Gene (bp)	No. of Exons	Strand	Chr Position	Mw (kDa)	pI	Instability Index	Aliphatic Index	Grand Average of Hydropathicity	Subcellular localization
1	SoARF1	Soffic.03G0037050	Soffic.03G0037050-1A	814	2445	4344	14	+	Chr03A	91.2	5.94	59.76	61.57	-0.642	Nuclear
2	SoARF2	Soffic.03G0037060	Soffic.03G0037060-1A	814	2445	4346	14	+	Chr03A	91.2	5.94	59.71	62.3	-0.633	Nuclear
3	SoARF3	Soffic.03G0036040	Soffic.03G0036040-2B	805	2418	4972	15	+	Chr03B	90.3	5.9	58.16	62.51	-0.652	Nuclear
4	SoARF4	Soffic.03G0036390	Soffic.03G0036390-1P	808	2427	5204	15	+	Chr03C	90.6	5.94	58.58	62.51	-0.639	Nuclear
5	SoARF5	Soffic.03G0037400	Soffic.03G0037400-4D	811	2436	4096	14	+	Chr03D	90.9	5.9	59.3	62.29	-0.647	Nuclear
6	SoARF6	Soffic.03G0038050	Soffic.03G0038050-2P	814	2445	4348	14	+	Chr03E	91.2	5.94	59.11	61.82	-0.64	Nuclear
7	SoARF7	Soffic.03G0037080	Soffic.03G0037080-6F	811	2436	4093	14	+	Chr03F	91.0	5.9	58.75	62.77	-0.636	Nuclear
8	SoARF8	Soffic.04G0004440	Soffic.04G0004440-1A	904	2715	5750	16	+	Chr04A	99.8	5.82	65.57	73.77	-0.428	Nuclear
9	SoARF9	Soffic.04G0004490	Soffic.04G0004490-1A	869	2610	4402	13	+	Chr04A	96.4	5.95	64.14	75.62	-0.384	Nuclear
10	SoARF10	Soffic.04G0002790	Soffic.04G0002790-2B	1153	3462	7337	14	–	Chr04B	128.2	6.06	59.8	78.7	-0.509	Chloroplast
11	SoARF11	Soffic.04G0004150	Soffic.04G0004150-2B	908	2727	5721	14	+	Chr04B	100.3	5.73	67.49	73.35	-0.437	Nuclear
12	SoARF12	Soffic.04G0017990	Soffic.04G0017990-2B	672	2019	4875	14	–	Chr04B	75.0	5.99	60.05	69.38	-0.555	Nuclear
13	SoARF13	Soffic.04G0002940	Soffic.04G0002940-3C	1153	3462	7837	15	–	Chr04C	128.0	5.96	59.39	78.45	-0.507	Chloroplast
14	SoARF14	Soffic.04G0004310	Soffic.04G0004310-3C	908	2727	5933	14	+	Chr04C	100.4	5.73	67.48	73.34	-0.438	Nuclear
15	SoARF15	Soffic.04G0003140	Soffic.04G0003140-4D	1148	3447	7097	14	–	Chr04D	127.5	6.01	59.75	78.7	-0.502	Chloroplast
16	SoARF16	Soffic.04G0004650	Soffic.04G0004650-4D	908	2727	5122	14	+	Chr04D	100.3	5.73	66.89	73.45	-0.434	Nuclear
17	SoARF17	Soffic.04G0028690	Soffic.04G0028690-1PD	771	2316	3633	8	–	Chr04D	84.9	5.54	65.13	68.17	-0.508	Nuclear
18	SoARF18	LAp.04E0004520	LAp.04E0004520	904	2715	5505	14	+	Chr04E	99.8	5.82	65.57	73.77	-0.428	Nuclear
19	SoARF19	LAp.04E0015420	LAp.04E0015420	672	2019	4875	14	–	Chr04E	75.0	5.99	60.05	69.38	-0.555	Nuclear
20	SoARF20	Soffic.04G0003710	Soffic.04G0003710-5F	909	2730	6015	16	+	Chr04F	100.5	5.78	67	73.05	-0.434	Nuclear
21	SoARF21	Soffic.04G0004850	Soffic.04G0004850-6G	908	2727	5769	14	+	Chr04G	100.3	5.73	67.39	73.45	-0.434	Nuclear
22	SoARF22	Soffic.04G0002910	Soffic.04G0002910-6H	1148	3447	7097	14	–	Chr04H	127.5	6.06	59.58	78.7	-0.502	Chloroplast
23	SoARF23	Soffic.04G0004380	Soffic.04G0004380-7H	908	2727	5838	14	+	Chr04H	100.3	5.73	67.49	73.24	-0.436	Nuclear
24	SoARF24	Soffic.05G0013390	Soffic.05G0013390-1A	857	2574	5855	16	–	Chr05A	94.0	6.29	54.21	62.05	-0.587	Nuclear
25	SoARF25	Soffic.05G0014560	Soffic.05G0014560-2B	857	2574	7908	16	–	Chr05B	93.9	6.29	54.39	61.49	-0.594	Nuclear
26	SoARF26	Soffic.05G0014100	Soffic.05G0014100-3C	850	2553	5859	16	–	Chr05C	93.4	6.36	54.43	61.66	-0.601	Nuclear
27	SoARF27	Soffic.05G0013410	Soffic.05G0013410-4D	857	2574	5887	16	–	Chr05D	94.0	6.25	54.76	62.51	-0.583	Nuclear
28	SoARF28	Soffic.05G0013520	Soffic.05G0013520-5E	857	2574	6157	15	–	Chr05E	93.9	6.29	54.16	61.49	-0.594	Nuclear
29	SoARF29	Soffic.05G0013340	Soffic.05G0013340-6G	857	2574	5882	16	–	Chr05G	94.0	6.25	54.3	61.83	-0.588	Nuclear
30	SoARF30	Soffic.05G0011550	Soffic.05G0011550-7H	857	2574	5813	16	–	Chr05H	94.1	6.38	54.27	61.83	-0.6	Nuclear
31	SoARF31	Soffic.06G0023610	Soffic.06G0023610-1A	946	2841	5026	13	–	Chr06A	104.0	5.82	62.02	68.67	-0.478	Nuclear
32	SoARF32	Soffic.06G0024730	Soffic.06G0024730-2B	947	2844	4916	13	–	Chr06B	104.3	5.77	61.53	69.21	-0.484	Nuclear
33	SoARF33	Soffic.06G0007800	Soffic.06G0007800-3C	661	1986	6556	14	+	Chr06C	73.2	5.95	59.37	70.08	-0.433	Nuclear
34	SoARF34	Soffic.06G0022780	Soffic.06G0022780-3C	947	2844	4918	13	–	Chr06C	104.2	5.87	61.65	68.9	-0.484	Nuclear
35	SoARF35	Soffic.06G0021990	Soffic.06G0021990-4D	947	2844	4921	13	–	Chr06D	104.3	5.73	61.7	69.1	-0.482	Nuclear
36	SoARF36	Soffic.06G0023200	Soffic.06G0023200-5E	947	2844	4920	13	+	Chr06E	104.3	5.77	61.81	69.42	-0.481	Nuclear
37	SoARF37	LAp.06F0008670	LAp.06F0008670	661	1986	6653	14	+	Chr06F	73.2	5.95	59.37	70.08	-0.433	Nuclear
38	SoARF38	Soffic.06G0021630	Soffic.06G0021630-6F	947	2844	7144	13	–	Chr06F	104.2	5.79	61.03	69.51	-0.47	Nuclear
39	SoARF39	Soffic.06G0024430	Soffic.06G0024430-7G	946	2841	4920	13	+	Chr06G	104.2	5.77	62.16	69.49	-0.482	Nuclear
40	SoARF40	LAp.06G0024610	LAp.06G0024610	947	2844	4920	13	–	Chr06G	104.3	5.77	61.81	69.42	-0.481	Nuclear
41	SoARF41	LAp.06H0021410	LAp.06H0021410	947	2844	4920	13	+	Chr06H	104.3	5.77	61.81	69.42	-0.481	Nuclear
42	SoARF42	Soffic.06G0021620	Soffic.06G0021620-8H	947	2844	4920	13	–	Chr06H	104.3	5.77	61.61	69.21	-0.484	Nuclear
43	SoARF43	Soffic.07G0019330	Soffic.07G0019330-1A	1099	3300	6688	13	–	Chr07A	121.8	6.03	57.98	75.7	-0.467	Nuclear
44	SoARF44	Soffic.07G0018870	Soffic.07G0018870-2B	1099	3300	6653	13	–	Chr07B	121.7	5.97	57.62	75.7	-0.46	Nuclear
45	SoARF45	Soffic.07G0018760	Soffic.07G0018760-3C	1099	3300	6737	13	–	Chr07C	121.6	6.21	57.84	75.6	-0.462	Nuclear
46	SoARF46	Soffic.07G0020210	Soffic.07G0020210-4D	1099	3300	6974	13	–	Chr07D	121.7	6.13	58.39	75.41	-0.471	Nuclear
47	SoARF47	Soffic.07G0018090	Soffic.07G0018090-5E	1099	3300	6882	14	–	Chr07E	121.5	6.08	57.31	75.15	-0.472	Nuclear
48	SoARF48	Soffic.07G0018550	Soffic.07G0018550-7G	1099	3300	6640	13	–	Chr07G	121.7	6.13	57.75	75.15	-0.472	Nuclear
49	SoARF49	Soffic.08G0009000	Soffic.08G0009000-7H	847	2544	6016	15	–	Chr08H	93.3	6.23	55.58	65.29	-0.595	Nuclear
50	SoARF50	Soffic.10G0005920	Soffic.10G0005920-1A	1053	3162	6121	13	+	Chr10A	116.7	6.15	70.78	79.79	-0.511	Nuclear
51	SoARF51	Soffic.10G0019420	Soffic.10G0019420-1A	921	2766	6335	14	–	Chr10A	102.0	5.92	68.83	74.1	-0.47	Nuclear
52	SoARF52	Soffic.10G0021640	Soffic.10G0021640-1A	1155	3468	8273	14	+	Chr10A	127.8	6.35	63.04	72.75	-0.557	Nuclear
53	SoARF53	Soffic.10G0007410	Soffic.10G0007410-2B	1053	3162	6587	13	+	Chr10B	116.8	6.37	69.39	80.62	-0.489	Nuclear
54	SoARF54	Soffic.10G0007330	Soffic.10G0007330-3C	1053	3162	6125	13	+	Chr10C	116.6	6.15	70.17	79.79	-0.509	Nuclear
55	SoARF55	Soffic.10G0020750	Soffic.10G0020750-3C	922	2769	7178	15	–	Chr10C	102.1	5.92	68.81	73.82	-0.479	Nuclear
56	SoARF56	Soffic.10G0020780	Soffic.10G0020780-1P	919	2760	5719	14	–	Chr10C	101.8	5.95	68.48	73.74	-0.474	Nuclear
57	SoARF57	Soffic.10G0007490	Soffic.10G0007490-4D	1053	3162	6125	13	+	Chr10D	116.6	6.15	69.75	79.79	-0.512	Nuclear
58	SoARF58	LAp.10D0020810	LAp.10D0020810	922	2769	6651	14	–	Chr10D	102.1	5.92	68.81	73.82	-0.479	Nuclear
59	SoARF59	Soffic.10G0023800	Soffic.10G0023800-1PD	1154	3465	8587	14	+	Chr10D	127.8	6.21	63.69	72.56	-0.564	Nuclear
60	SoARF60	Soffic.10G0006680	Soffic.10G0006680-1P	893	2682	4929	8	+	Chr10E	99.3	6.4	71.3	79.78	-0.542	Cytoplasm
61	SoARF61	Soffic.10G0021240	Soffic.10G0021240-3PE	921	2766	5653	14	–	Chr10E	101.9	5.92	68.71	74.12	-0.463	Nuclear
62	SoARF62	LAp.10E0021250	LAp.10E0021250	922	2769	6643	14	–	Chr10E	102.1	5.92	68.81	73.82	-0.479	Nuclear
63	SoARF63	Soffic.10G0023830	Soffic.10G0023830-5E	1154	3465	8263	14	+	Chr10E	127.6	6.27	63.25	72.65	-0.559	Nuclear
64	SoARF64	Soffic.10G0005030	Soffic.10G0005030-6F	981	2946	6118	11	+	Chr10F	108.7	6.34	70.32	80.76	-0.504	Nuclear
65	SoARF65	Soffic.10G0019400	Soffic.10G0019400-5PF	919	2760	7254	14	–	Chr10F	101.8	5.92	69.15	73.32	-0.478	Nuclear
66	SoARF66	Soffic.10G0021930	Soffic.10G0021930-6F	1155	3468	8381	14	+	Chr10F	127.8	6.19	64.2	72.68	-0.561	Nuclear
67	SoARF67	LAp.10G0006440	LAp.10G0006440	981	2946	6118	11	+	Chr10G	108.7	6.34	70.32	80.76	-0.504	Nuclear
68	SoARF68	Soffic.10G0021010	Soffic.10G0021010-6PG	923	2772	6362	14	–	Chr10G	102.2	5.92	68.45	73.74	-0.476	Nuclear
69	SoARF69	Soffic.10G0023460	Soffic.10G0023460-7G	1155	3468	8701	14	+	Chr10G	127.8	6.37	63.9	72.75	-0.558	Nuclear
70	SoARF70	Soffic.10G0007910	Soffic.10G0007910-7H	1052	3159	6257	13	+	Chr10H	116.6	6.25	69.9	79.4	-0.507	Nuclear
71	SoARF71	LAp.10H0024350	LAp.10H0024350	857	2574	5876	16	–	Chr10H	94.0	6.25	54.76	62.51	-0.583	Nuclear
72	SoARF72	LAp.00010190	LAp.00010190	1148	3447	7096	14	+	utg000397l_926000_935999	127.5	6.06	59.58	78.7	-0.502	Chloroplast
73	SoARF73	LAp.00043220	LAp.00043220	1148	3447	7339	14	+	utg002331l_93000_110999	127.5	6.06	59.58	78.7	-0.502	Chloroplast

### 3.2 Genome-wide orthologs and ARF gene cluster analysis

**Figure 1:**
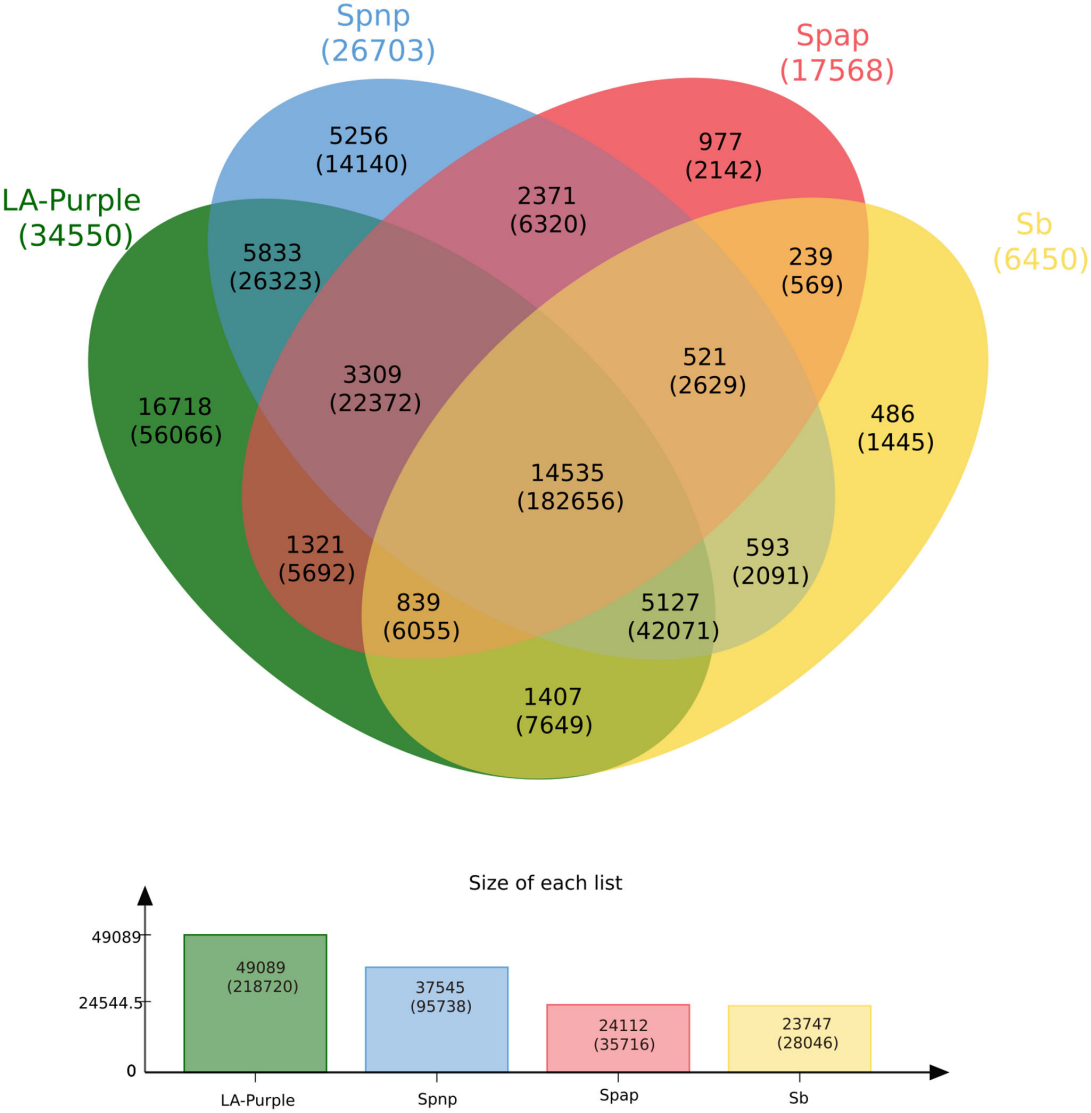
Orthologous gene clusters among *S. officinarum* LA-Purple, *S. spontaneum* AP85-441 (Spap), *S. spontaneum* Np-X (Spnp), and *S. bicolor* (Sb). The number in each sector of the diagram indicates the number of homologous clusters and the numbers in parentheses indicate the total number of genes contained within the associated clusters. The numbers in parentheses below the species names indicate the number of species-specific singletons (genes with no homologs).

Comparative genome analysis was conducted to explore the genes pace of *S. officnarum* LA-Purple, *S. spontaneum* AP85-441 (Spap), *S. spontaneum* Np-X (Spnp), and *S. bicolor* (Sb) (**Figure 1**). The maximum clusters of 49089 (218,720 proteins) were recorded in *S. officnarum* LA-Purple, followed by 37545 (957,38 proteins) clusters in *S. spontaneum* Np-X, and then 24112 (357,16 proteins) clusters in *S. spontaneum* AP85–441 and 23747 (280,46 proteins) clusters in *S. bicolor*. Meanwhile, a total of 14,535 orthologous clusters (182,656 proteins) were common in these four genomes. In addition, 3309 clusters (22372 proteins) were found to be unique to LA-Purple, Spnp and Spap genomes, while 593 clusters (2091 proteins) were identified as specific to both *S. bicolor* and Spnp species. The higher number of 34550–26703 singletons was observed in the LA-Purple and Spnp genomes, while the lower number of 6450 singletons was found in the *S. bicolor* genome (**Figure 1**). These results suggest the gene expansion and potential functional diversification in sugarcane. Furthermore, orthologous clusters among ARF gene families in four genomes including LA-Purple, AP85-441 (Spap), Np-X (Spnp), and *S. bicolor* (Sb) were compared in order to identify orthologous clusters and overlapping regions. In contrast *S. bicolor* contained relatively fewer unique gene families, indicating a more conserved genome structure. Overall, 7 (73 proteins), 27 (27 proteins), 10 (17 proteins), and 18 (25 proteins) orthologous clusters were found in *S. officnarum* LA-Purple, *S. spontaneum* AP85-441 (Spap), *S. spontaneum* Np-X (Spnp), and *S. bicolor* (Sb) respectively (**Supplementary Figure 1**). In contrast, five clusters (77 proteins) were common to the LA-Purple, *S. bicolor* and AP85–441 genomes, while two clusters (21 proteins) were common to the LA-Purple and *S. bicolor* genomes. In addition, five clusters (10 proteins) were specific to AP85–441 and *S. bicolor*, while six clusters (7 proteins) and 27 clusters (27 proteins) were uniquely specific to *S. bicolor* and Np-X genomes respectively (**Supplementary Figure 1**).

### 3.3 Evolutionary relationships of *ARF* genes across diverse plant species

**Figure 2:**
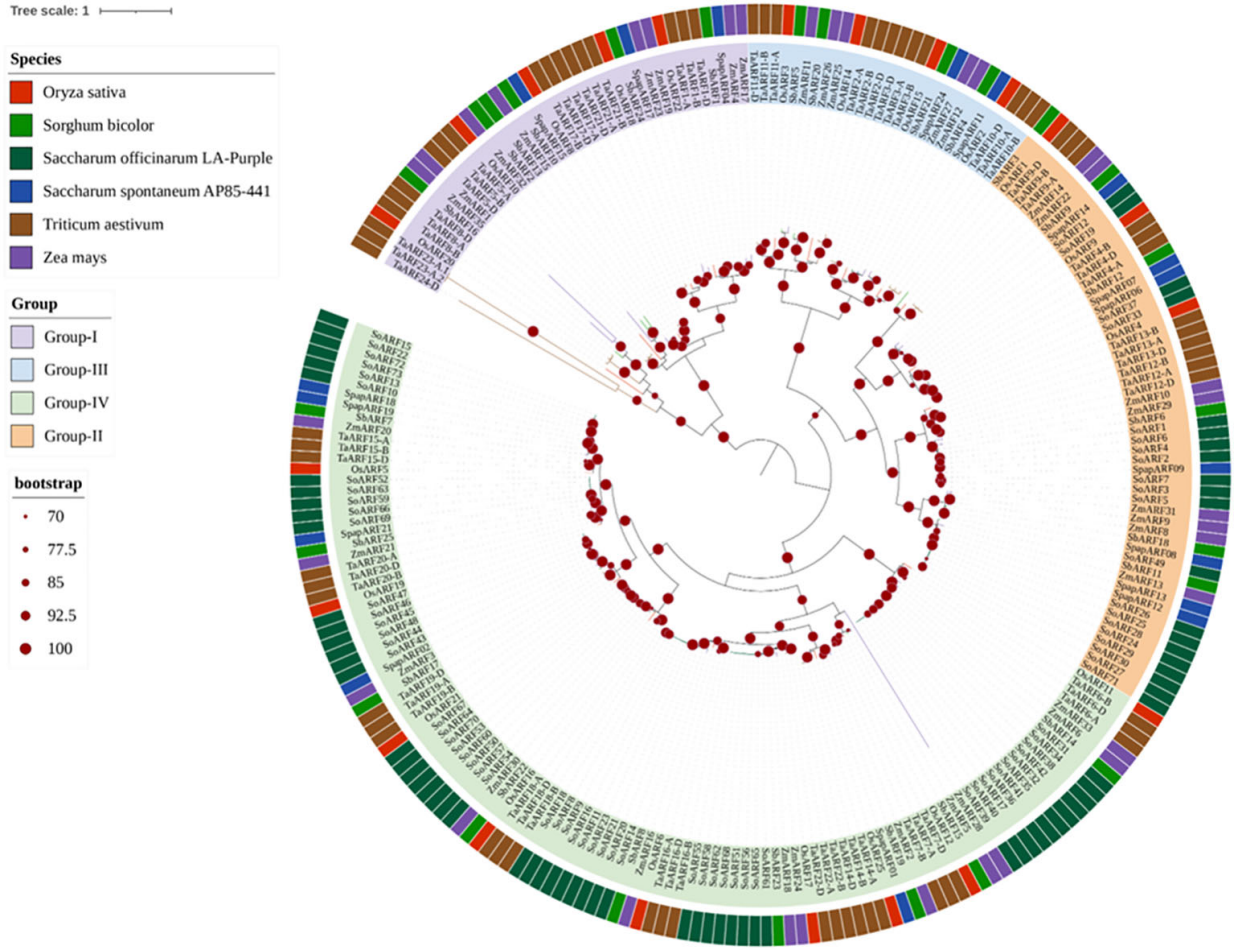
Phylogenetic tree of ARF family genes from *S. officinarum* LA-Purple, *S. spontaneum* clone AP85-441 (Spap), *S. bicolor* (Sb), *T. aestivum* (Ta), *O. sativa* (Os), and *Z. mays* (Zm). The tree was constructed using the maximum likelihood (ML) method with 1,000 bootstrap replicates implemented in IQ-TREE2. Four phylogenetic groups (I, II, III, and IV) are distinguished by different colored backgrounds.

Phylogenetic tree of *ARF* family genes from six monocot species such as *S. officinarum* LA-Purple, *S. spontaneum* clone AP85-441 (Spap), *S. bicolor* (Sb), *T. aestivum* (Ta), *O. sativa* (Os), and *Z. mays* (Zm) was constructed and represented in **Figure 2**. On comparison, phylogenetic analysis showed four major groups (I, II, III, and IV). Group IV was the largest one (115 ARF members), while in the smallest group III only 27 members were reported. Majority of the SoARF members were found in the clade IV (40), followed by clade II (33). There was no member in the clade I and III. Many sugarcane *SoARFs* cluster with genes from *S. spontaneum*, *S. bicolor*, maize, and rice which indicate the conserved gene family in the monocot species.

### 3.4 Phylogenetic, structural characterization, conserved domains, and motifs analysis of *SoARF* genes

**Figure 3:**
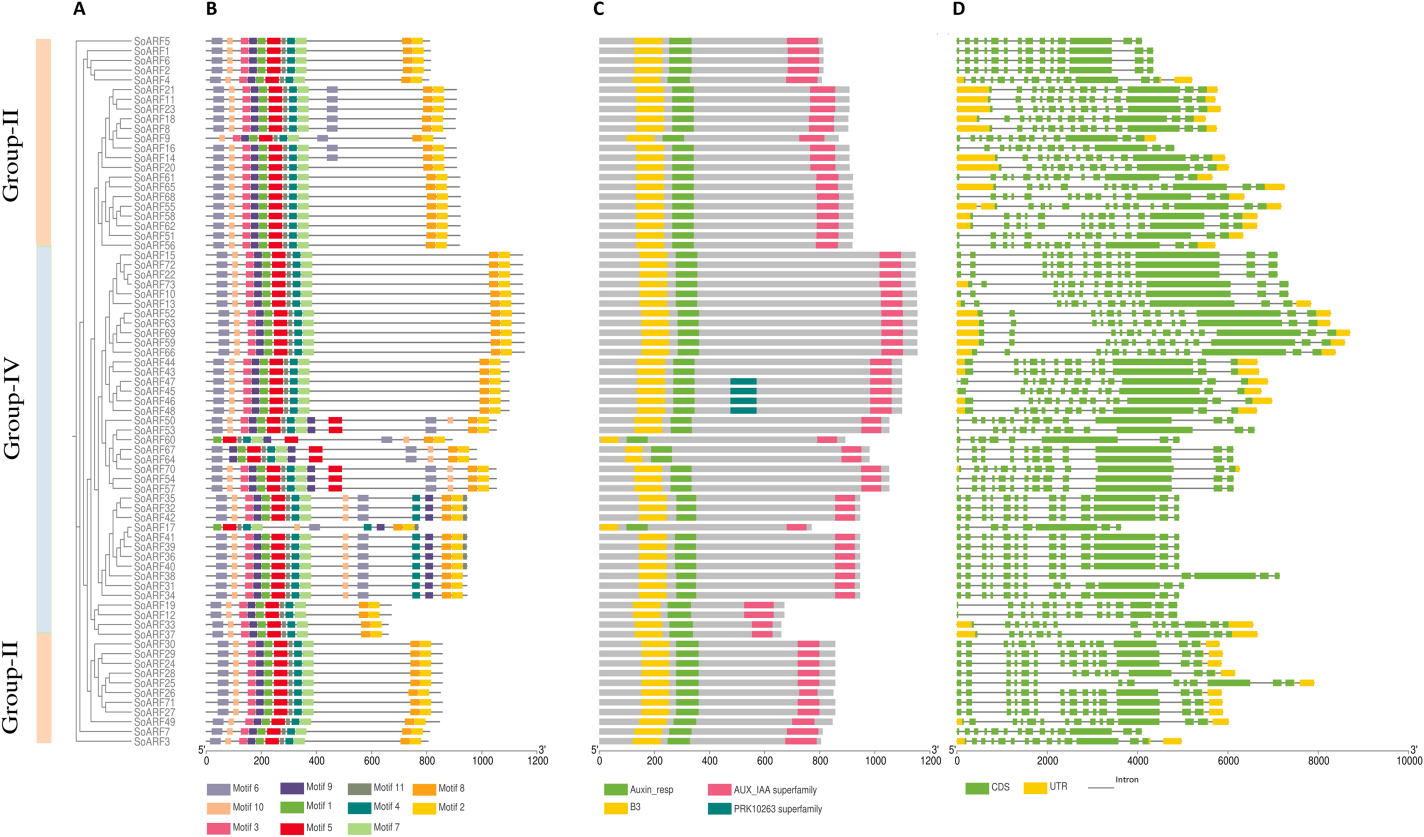
Structural characterization of ARF genes in *S. officinarum* LA-Purple. **(A)** Phylogenetic tree **(B)** Conserved motifs, **(C)** conserved domains, and **(D)** exon–intron structures of ARF genes. **(A)** Groups II and IV correspond to the phylogenetic groups (I–IV) shown in **Figure 2**. **(B)** Conserved motifs. Motifs were identified using MEME (version 5.0.2). Each distinct motif is represented by a colored box, numbered 1 to 11 at the bottom. **(C)** Conserved domains. Functional domains were annotated based on gene model annotations and NCBI CDD-batch search results. **(D)** Exon–intron structures. Exons and introns are depicted as boxes and lines, respectively. Exon, intron, and untranslated region (UTR) lengths are indicated on the bottom scale. Protein lengths (in amino acids) are estimated using the scale at the bottom of panels **(A)** and **(B)**; gene lengths (in base pairs) are estimated using the scale at the bottom of panel **(C)**. B3: B3 DNA-binding domain, Auxin-resp: ARF (auxin response factor) domain, AUX/IAA: C-terminal dimerization domain, PRK10263 superfamily: Conserved protein domain family.

All the identified 73 *SoARF* genes were subjected to phylogenetic analysis. All the *SoARF* family genes were distributed into two groups (II and IV). Group IV contains the 40 SoARF members while the group II contains 33 members (**Figure 3A**). There were 11 conserved motifs were reported in all the *SoARF* genes based on the MEME results. Most SoARF members in Group IV exhibit duplication of certain motifs in their protein sequences. In contrast, seven SoARF members in Group II possess an additional Motif 6, while eight members in Group IV specifically contain an extra Motif 5 in their protein sequences (**Figure 3B**). Auxin response, B3, and AUX/IAA superfamily were conserved among all the 73 SoARF members, while SoARF45–48 also have extra domain PRK10263 superfamily (**Figure 3C** and **Supplementary Figure 2**). To understand more deeply about the conservation of *SoARF* family genes, we examined the gene structure (**Figure 3D**). Every one of the 73 *SoARF* genes has both 5’ and 3’ UTR regions. According to the gene structure analysis, the *SoARF* family genes in various evolutionary clades have remarkably altered exon-intron structures. Group IV shows the longest exon-introns as compared to group II. While the number of exons varied between 8-16. Interestingly, the number and position of introns and exons was largely consistent in the current study (**Figure 3D**).

### 3.5 Prediction of *Cis*-regulatory elements

**Figure 4:**
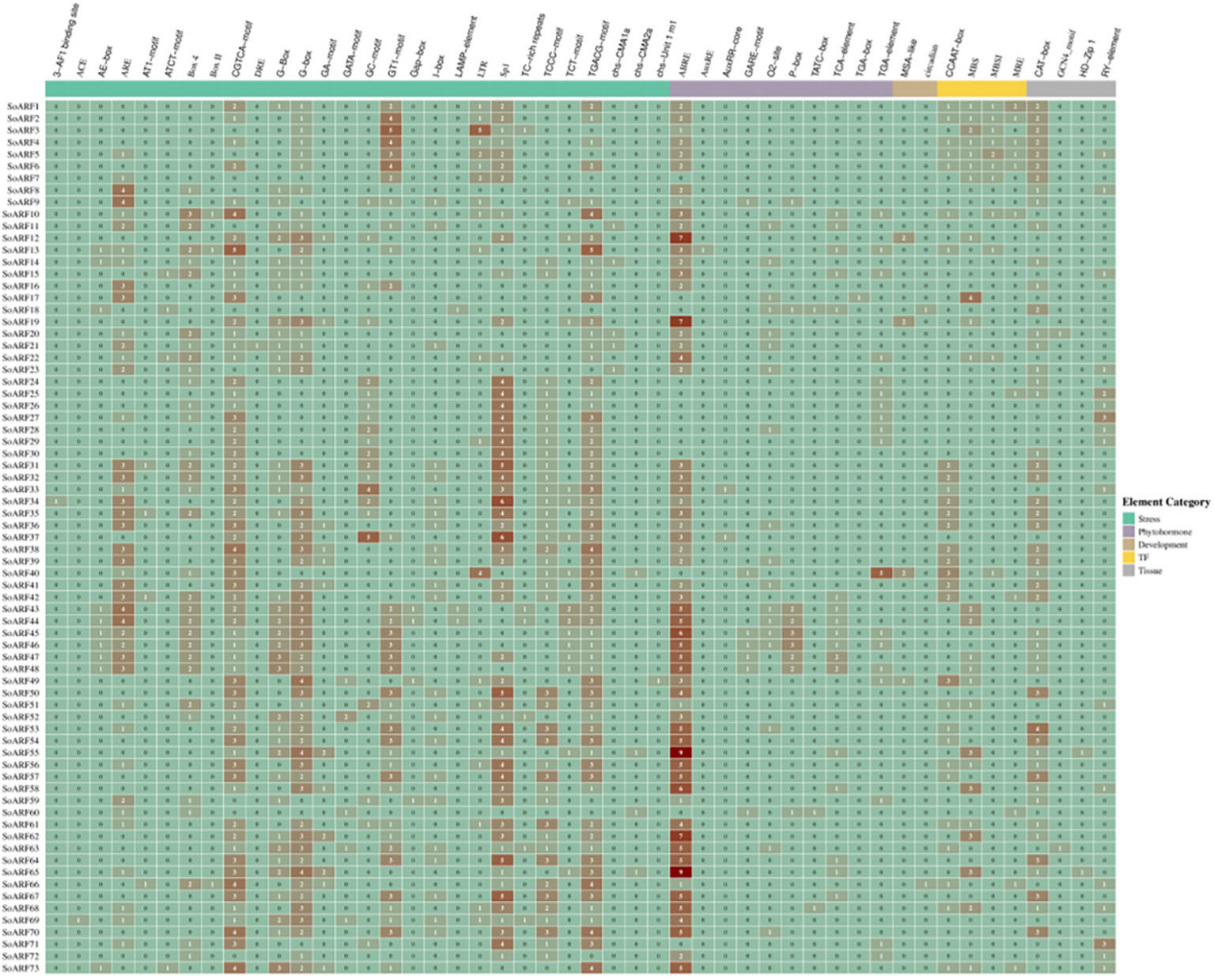
Heatmap of cis-regulatory elements in ARF promoters from *S. officinarum* LA-Purple. The cis-regulatory elements were identified in the 1.5 kb upstream promoter regions of ARF genes and classified into five categories: Stress, Phytohormone, Development, TF, and Tissue. Numbers in the cells indicate the cis-element count.

To clarify the functions of *SoARF* genes in biotic and abiotic stress responses, a *cis*-regulatory elements study of the 1.5 kb upstream promoter regions was carried out (**Figure 4**). Analysis revealed a diverse and uneven distribution of regulatory motifs across the promoter regions of *SoARF* genes, indicating the complex transcriptional control within the gene family. A total of 49 different types of *cis*-elements associated with biotic and abiotic stresses were found within the 1.5 kb promoter region of 73 *SoARF* genes. These elements were further categorized into five categories: stress, phytohormone, development, TF, and tissue-related (**Figure 4**). A wide range of elements associated with phytohormones responsiveness, particularly auxin-related motifs were detected across most genes, supporting their expected role in auxin signaling pathways. Additionally, several stress responsive elements (ARE, MBS, LTR, TC rich) such as those related to anaerobic induction and drought responsiveness (MBS, ABRE), were selectively enriched in specific genes, suggesting potential functional specialization in stress adaptation. The presence of light responsive (G-box, Box4, TCT-motif, GT1 motif) and developmental related elements (CAT box, O2 site, RY element) further highlights the involvement of *SoARF* genes in multiple physiological processes (**Figure 4**).

### 3.6 Chromosomal localization, gene duplication, and synteny analysis of *SoARF* genes

**Figure 5:**
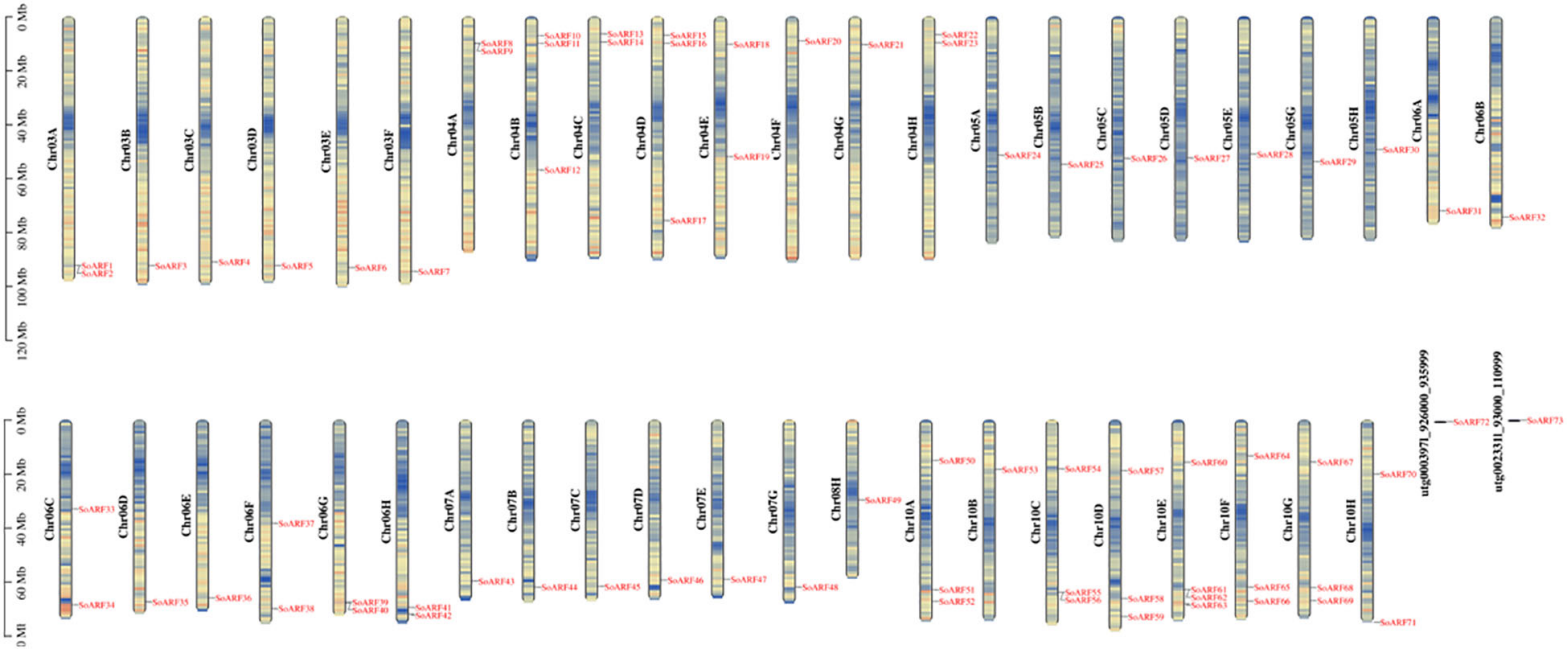
Distribution of *SoARF* genes on chromosomes of *S. officinarum* LA-Purple. Chromosome colors represent gene density, calculated with non-overlapping windows of 1 Mb.

The distribution of *SoARF* genes in the polyploid genome of *S. officinarum* LA-Purple was presented in **Figure 5**. The chromosomal localization analysis revealed that *SoARF* genes are widely but unevenly distributed across the chromosomes. A total of 73 genes were mapped to multiple chromosomes, with maximum four genes presented on chromosome 10E followed by three on chromosomes 4B, 4D, 4E, 10A, 10C, 10D, 10F, and 10G, indicating a non-random distribution pattern. Some genes were observed in close physical proximity on the same chromosome, suggesting the occurrence of tandem duplication. Additionally, the presence of homologous genes across different chromosomal segments and sub-genomes points towards the role of segmental or whole genome duplication in the expansion of the *SoARF* gene family (**Figure 5**).

**Figure 6:**
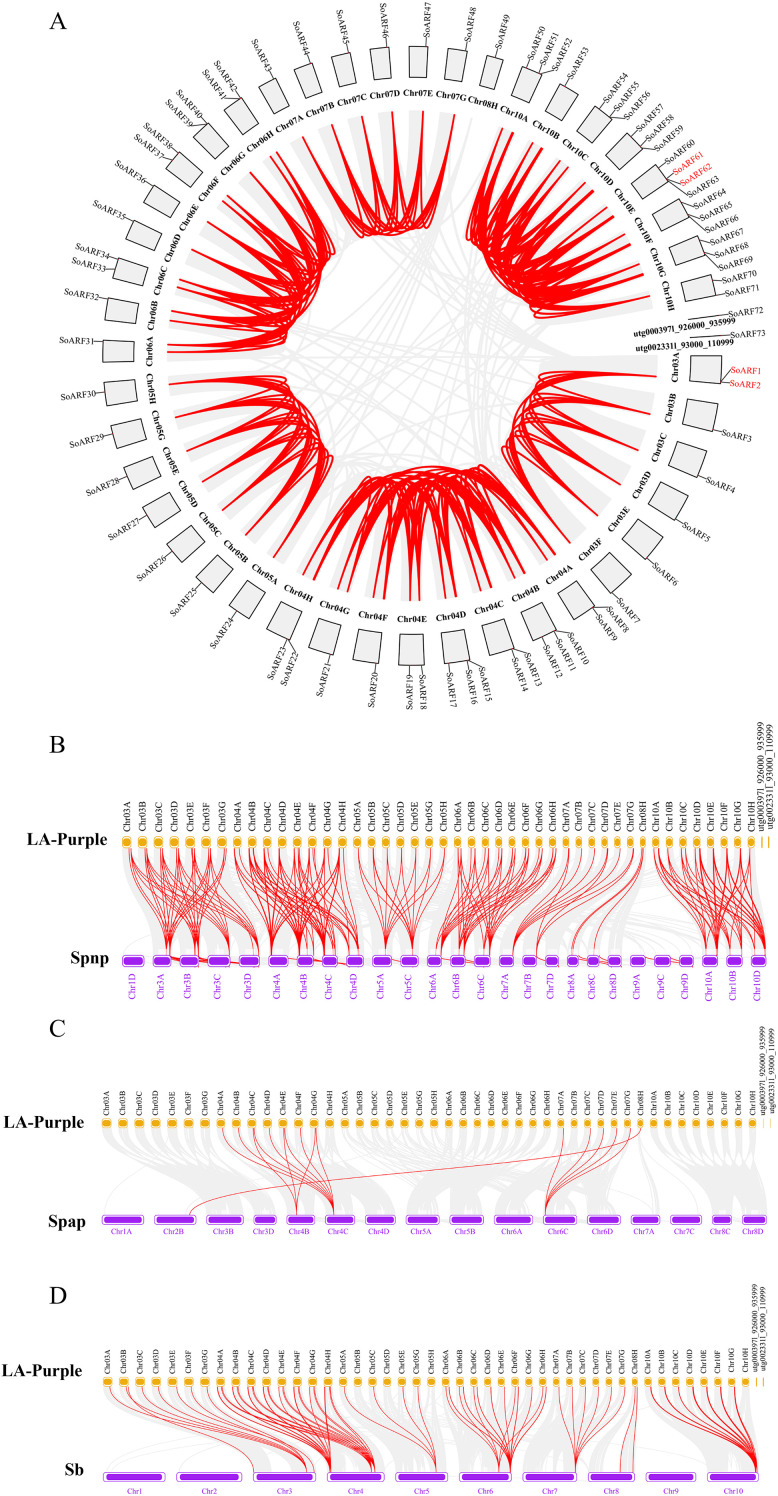
Genomic distribution and syntenic relationships of ARF genes. **(A)** Circos plot showing the distribution of duplicated ARF genes on chromosomes of *S. officinarum* LA-Purple. Gray ribbons represent collinear relationships among genome-wide blocks, while red ribbons indicate ARF paralogs. Genes highlighted in red denote segmentally duplicated events. **(B–D)** Collinearity analysis of ARF genes between *S. officinarum* LA-Purple and three related species: **(B)** *S. spontaneum* Np-X (Spnp), **(C)** *S. spontaneum* AP85-441 (Spap), and **(D)** *S. bicolor* (Sb). Red lines represent syntenic ARF gene pairs, whereas gray lines in the background indicate all orthologous gene pairs across the genomes.

To further investigate the expansion mechanism of the *SoARF* gene family, duplication and collinearity analysis were performed (**Figure 6** and **Supplementary Table 2**). The collinearity plot demonstrated that extensive inter chromosomal syntenic relationships, with numerous duplicated gene pairs connected across different chromosomes. This pattern indicates that segmental duplication and whole genome duplication have played a dominant role in the expansion of gene family. In contrast, relatively less intra-chromosomal duplication events were observed, suggesting a limited contribution of tandem duplication. Overall, these findings suggest that polyploidization has significantly influenced the structural organization of the *SoARF* genes (**Figure 6A**). Comparative synteny analysis with related species including *S. spontaneum* Np-X (Spnp), *S. spontaneum* AP85-441 (Spap), and *S. bicolor*, revealed conserved orthologous relationships for several *SoARF* genes. Multiple *SoARF* genes exhibited one-to-many syntenic correspondences with homologs in these species, reflecting gene expansion and retention following duplication events (**Figures 6B–D**). A total of 62 *SoARF* family members from *S. officinarum* LA-Purple shared syntenic association with *S. spontaneum* Np-X, seven with *S. spontaneum* AP85-441, and 46 with *S. bicolor*. Conversely, some *SoARF* genes lacked identifiable syntenic counterparts, which may indicate lineage-specific gene loss or functional divergence (**Figure 6** and **Supplementary Table 2**).

### 3.7 Identification of microRNAs and PPI

For better understands the roles of miRNAs in controling the post-transcriptional modifications of *ARF* genes, the 73 *SoARF* CDS sequences were compared to known miRNAs sequences from *Z. mays* using the psRNATarget web services (**Supplementary Figure 3** and **Supplementary Table 3**). A total of 618 miRNAs network were predicted to target all the 73 *SoARF* genes. These miRNAs were further categorized into 70 unique classes, belonging to 20 distinct miRNA families. Notably, members of the miRNA160 and miRNA167 families exhibited high connectivity, targeting multiple *SoARF* genes and suggesting their central role in auxin signaling regulation. In addition, other miRNAs including miRNA169, miRNA171, miRNA396, and miRNA444 were found to target specific *SoARFs*, indicating additional layers of regulatory complexity. *SoARF11*, *SoARF14* and *SoARF16* were targeted by 25 miRNAs each (**Supplementary Figure 3** and **Supplementary Table 3**).

**Figure 7:**
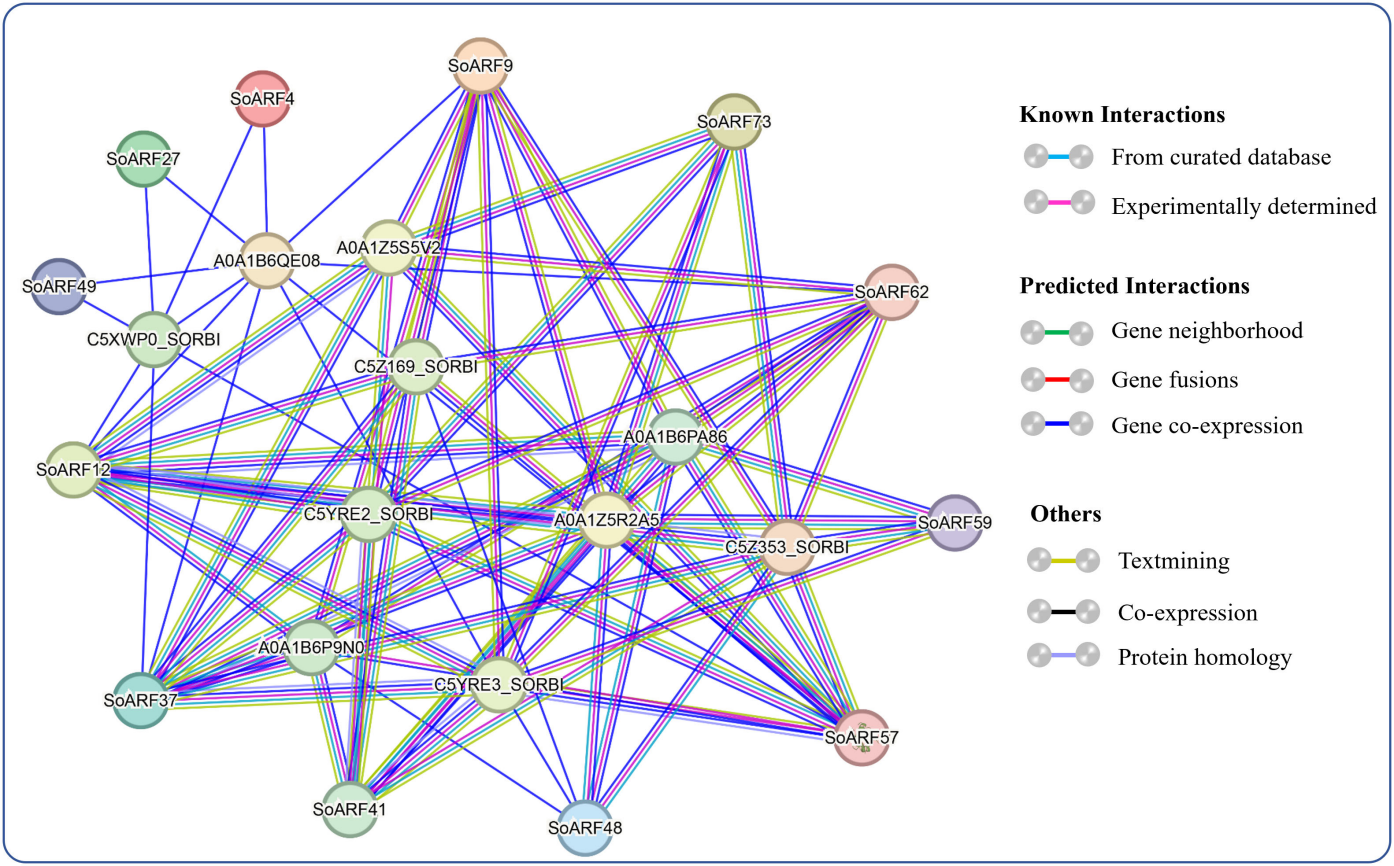
Predicted protein–protein interaction network of *S. officinarum* LA-Purple ARF proteins. Interactions were inferred from homologous pairs with *S. bicolor* using STRING. Only pairs with >90% sequence identity and an interaction score >0.4 are shown. Edge colors indicate interaction types; node colors reflect interaction confidence. **Supplementary Table 4** contains full network details.

Proteins in the cell do not function independently but work together as a part of complex biological systems. In the current study, the PPI network of SoARF proteins from *S. officinarum* LA-Purple and their orthologs in *S. bicolor* was constructed using STRING database (v12.0). SoARF proteins with >90% sequence identity and an interaction score >0.4 were used to generate PPI network (**Figure 7** and **Supplementary Table 4**). 71 proteins showed interactions among them. C5Z169_SORBI, C5YRE2_SORBBI, and C5Z353 were in the middle and showed interactions with SoARF59, SoARF62, SoARF57, SoARF12, SoARF37, SoARF9, and SoARF73 (**Figure 7** and **Supplementary Table 4**).

### 3.8 Expression analysis of *SoARF* genes under phytohormones treatment

**Figure 8:**
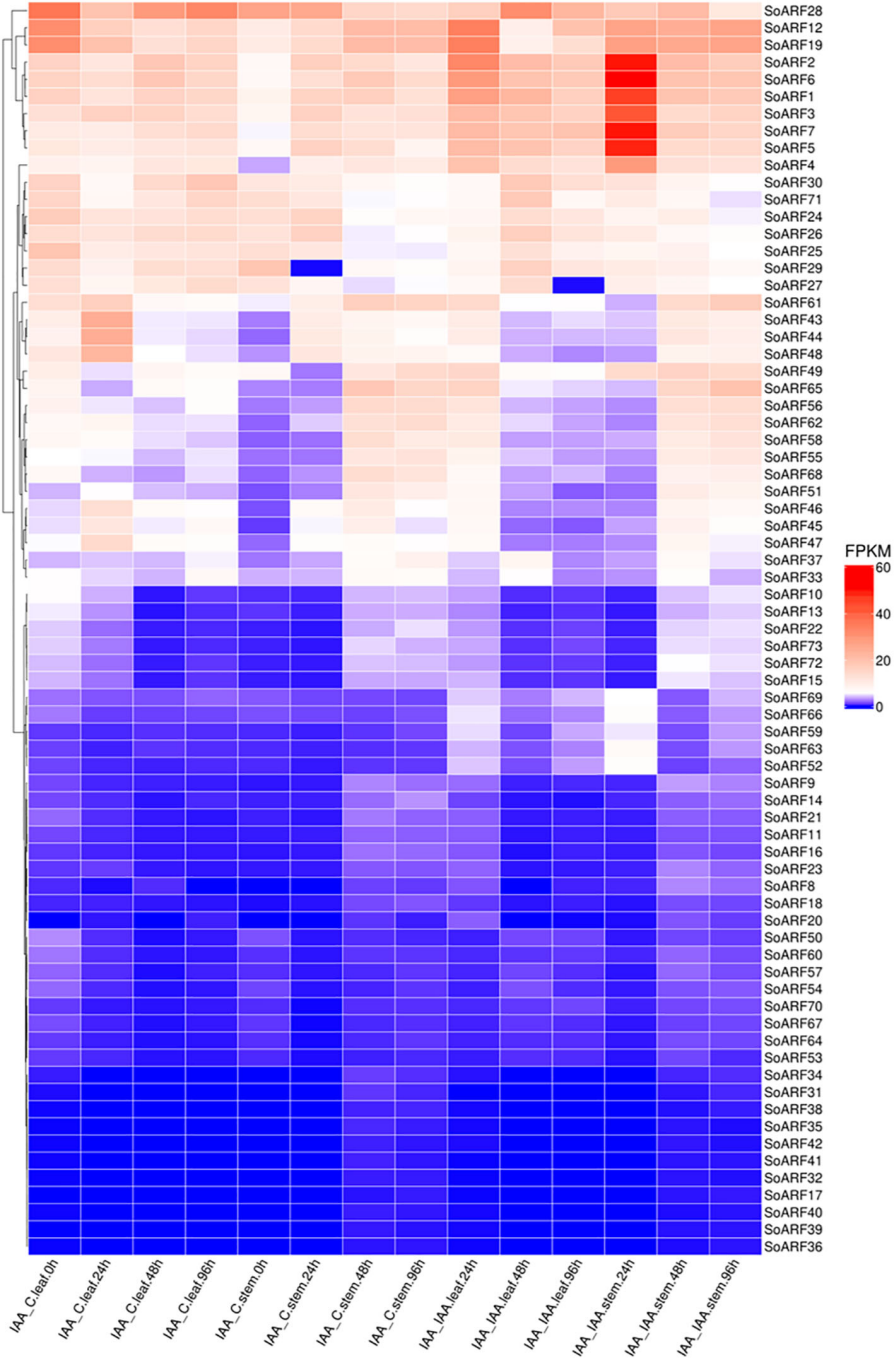
Expression profiles of *SoARF* genes in *S. officinarum* LA-Purple based on FPKM values. Expression dynamics of *SoARF* genes in seedling leaves and stems following Indole-3-acetic acid (IAA) treatment. Seedlings were treated with IAA for 24, 48, and 96 h, with untreated samples at corresponding time points serving as controls (0 h-CK). Details of the expression levels are highlighted in **Supplementary Table 5**. Differential expression was defined using |log_2_FoldChange| > 1 and *p* < 0.05.

Transcript profiles of all the 73 *SoARF* genes were assessed under differ different time points 0, 24, 48, and 96 h from the seedling leaves and stems following Indole-3-acetic acid (IAA) treatment (**Figure 8** and **Supplementary Table 5**). Small group of genes such as *SoARF12*, *SoARF28, SoARF19, SoARF2, SoARF1, SoARF3*, and *SoARF7* shows consistently high expression, particularly under certain IAA treatments, indicating they may play key role in auxin response. In contrast the genes such as *SoARF36*, *SoARF39, SoARF40, SoARF17, SoARF32, SoARF41*, and *SoARF42* were not induced or showed weaker expression, while some of the genes showed temporal expressions across different treatment time points. Furthermore, several genes exhibited tissue-specific and temporal expression patterns. For instance, *SoARF51*, *SoARF55*, *SoARF56*, *SoARF58*, *SoARF62*, and *SoARF68* were significantly upregulated in stem tissue at 48- and 96 h post-treatment (hpt), while showing downregulation in leaf tissue at the same time points. Similarly, *SoARF33*, *SoARF37*, *SoARF45*, *SoARF46*, and *SoARF47* displayed distinct expression profiles in stems and leaves across different treatment time points relative to their respective controls (**Figure 8** and **Supplementary Table 5**). These results also indicate that *SoARF* genes exhibit tissue- and time-specific expression at certain time points, suggesting their differential roles in regulating stem and leaf responses under the IAA treatment conditions.

**Figure 9:**
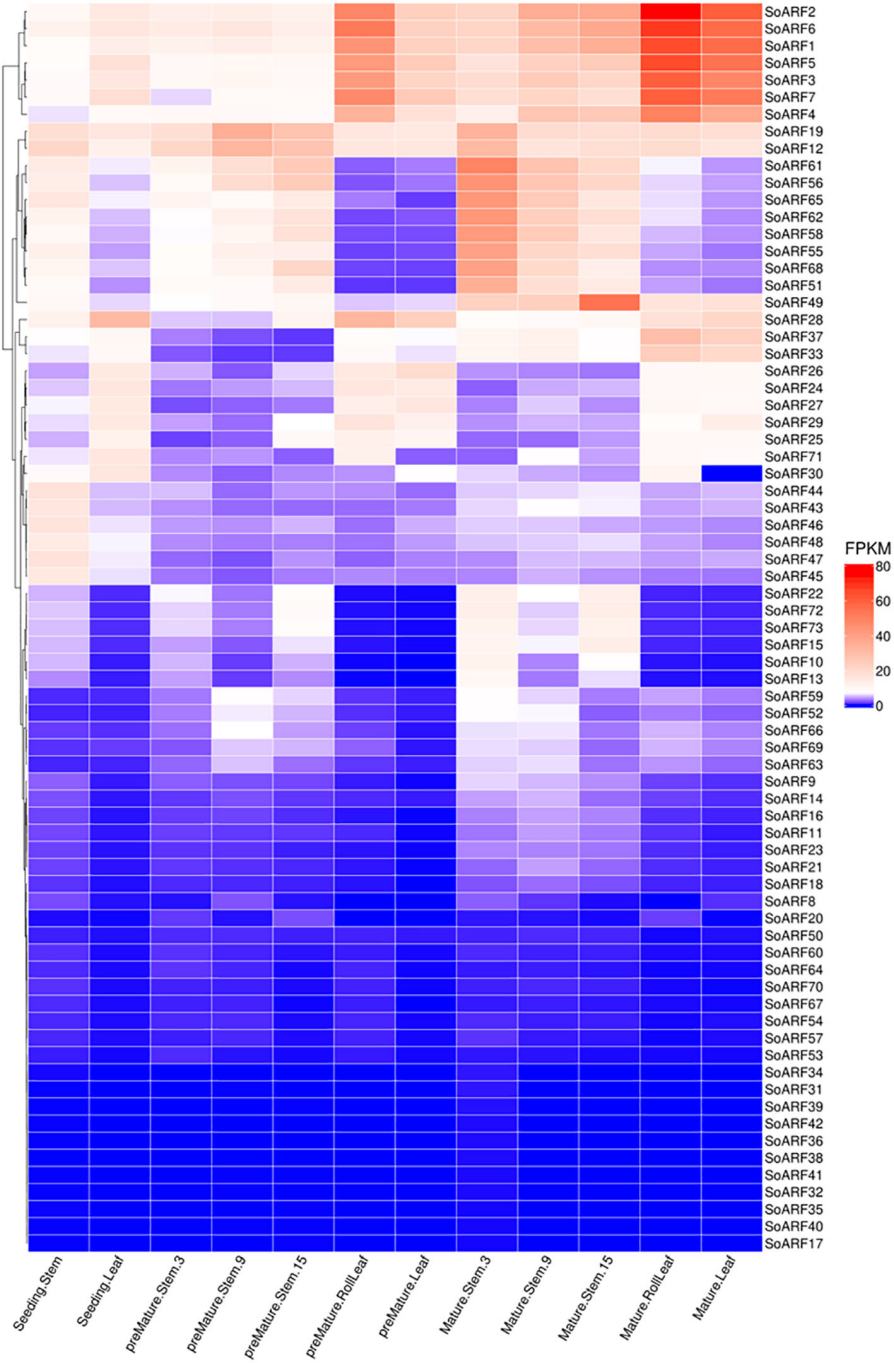
Expression profiles of *SoARF* genes in *S. officinarum* LA-Purple based on FPKM values. Expression patterns of *SoARF* genes across different tissues and developmental stages. Samples included leaf and stem at the seedling stage (35 days), and rolled leaf, leaf, and three stem nodes (stem3, stem9, stem15) at the pre-mature (9 months) and mature (12 months) stages. Details of the expression levels are highlighted in **Supplementary Table 6**. Differential expression was defined using |log2FoldChange| > 1 and *p* < 0.05.

We further analyzed the expression profiling of *SoARF* genes under different development stages including leaf and stem from seeding stage (35 days) SES-208; the roll leaf, leaf, top immature stem node (stem3), middle premature stem node (stem9), base mature stem node (stem15) from pre-mature stage (9 months) and mature stage (12 months) were used (**Figure 9** and **Supplementary Table 6**). The expression levels revealed substantial variation among *SoARF* family members, indicating strong spatial and developmental specificity. A subset of genes, including *SoARF2*, *SoARF6, SoARF1, SoARF5, SoARF3, SoARF7*, and *SoARF4* displayed relatively high expression, particularly in mature leaf and mature rolled leaf tissues, suggesting important role in leaf development and auxin mediated regulations. Conversely, 28 *SoARF* genes were significantly downregulated across all examined plant tissues, indicating potential negative regulatory roles in plant development. Additionally, *SoARF10*, *SoARF13*, *SoARF15*, *SoARF22*, *SoARF72* and *SoARF73* distinct tissue-specific expression patterns, with strong upregulation in stem tissues and pronounced downregulation in leaf tissues, suggesting specialized functions in growth and development. Moreover, a few genes, including *SoARF24*, *SoARF26*, *SoARF27*, *SoARF29*, *SoARF33*, and *SoARF37*, were highly upregulated in leaf tissues while being significantly downregulated in stem tissues, further emphasizing the tissue-specific roles of *SoARF* genes in plant development (**Figure 9** and **Supplementary Table 6**).

### 3.9 Tissue-dependent transcriptional responses of *SoARF* genes under IAA treatment

**Figure 10:**
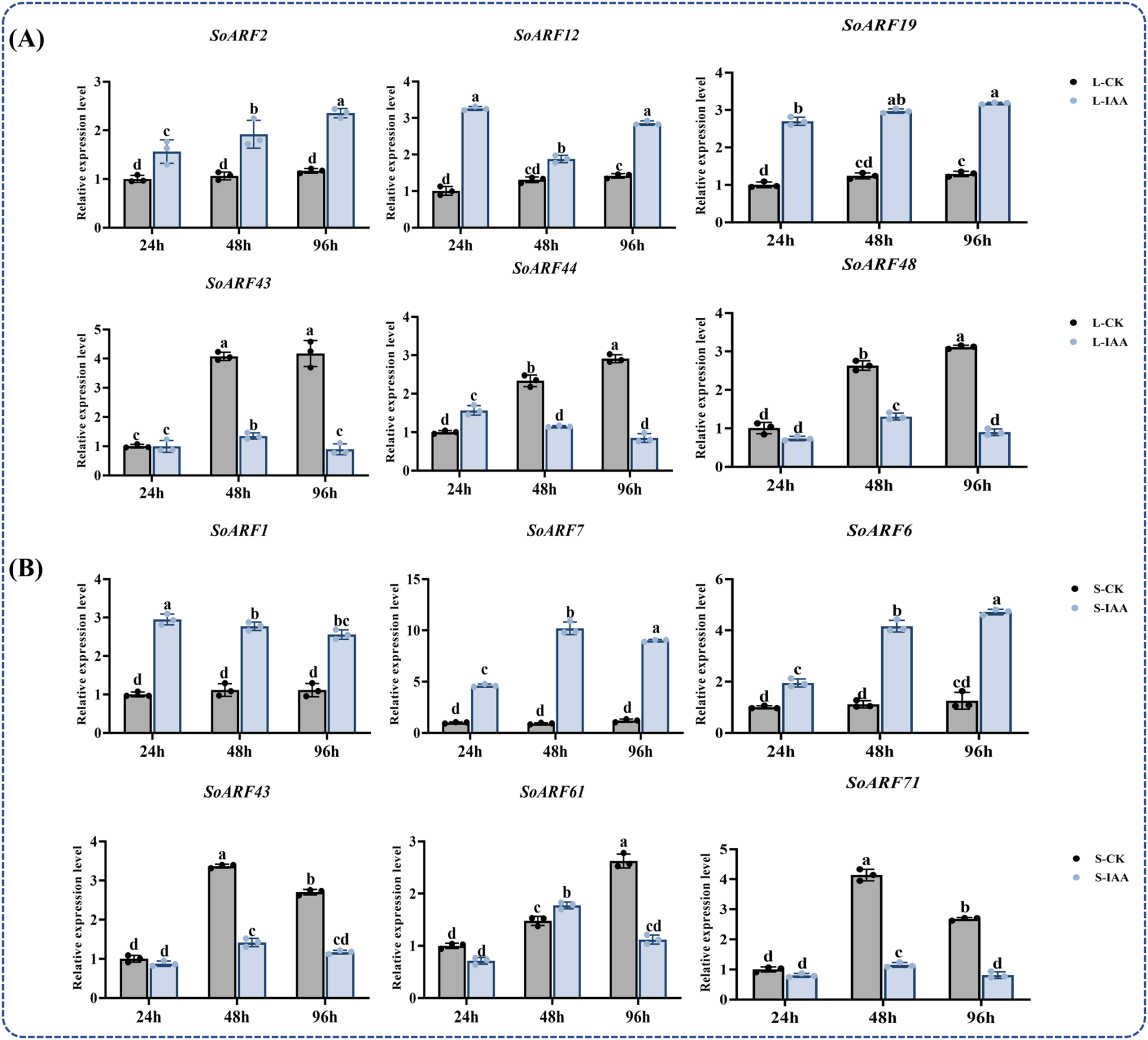
RT-qPCR based expression profiling of *SoARF* family members in Leaf tissue **(A)** and Stem tissue **(B)** of sugarcane cultivar (GT58) under IAA treatment. Tissue samples were collected at 24, 48, and 96 h post-treatment (hpt). Mock-treated plants (CK) sprayed with distilled water served as control. Vertical bars represent the mean ± standard error (three replicates). Different letters (a, b, c, d) on the bar represent significant differences among treatments. Statistical significance was determined using Student’s t-test; *, **, and *** indicate *p* < 0.05, *p* ≤ 0.01, and *p* ≤ 0.001, respectively. L: Leaf tissue, S: Stem tissue.

To investigate the auxin responsiveness and tissue-specific regulatory roles of *SoARF* genes, their expression patterns were analyzed in leaves and stems under IAA treatment at 24 h, 48 h, and 96 h (**Figure 10**). In leaves, *SoARF* genes exhibited moderate but distinct transcriptional responses. *SoARF2*, *SoARF12*, and *SoARF19* were consistently induced by IAA across all time points, showing a gradual increase and reaching peak expression at 96 h, suggesting their roles as auxin-inducible regulators. In contrast, *SoARF43*, *SoARF44*, and *SoARF48* showed significantly higher expression under control conditions but were markedly repressed upon IAA treatment, indicating potential involvement in auxin-mediated negative regulation or feedback control. Overall, the magnitude of transcriptional changes in leaves was relatively limited, generally within a 2–3 fold range, reflecting a finely tuned response to auxin (**Figure 10A**).

In comparison, stem tissues displayed more pronounced and dynamic transcriptional responses. *SoARF1*, *SoARF6*, and *SoARF7* were strongly induced by IAA at all examined time points, with SoARF7 showing a particularly dramatic increase, peaking at 48 h and maintaining high expression at 96 h, indicative of a highly sensitive auxin-responsive gene. *SoARF6* exhibited a continuous upregulation trend, while *SoARF1* showed an early induction followed by a slight decline but remained significantly higher than the control. Conversely, *SoARF43* and *SoARF71* were highly expressed under control conditions but significantly suppressed by IAA treatment, especially at 48 h, suggesting negative regulation by auxin in stem tissues. In addition, *SoARF61* displayed a distinct pattern, with expression increasing over time under both treatments but remaining relatively higher under control conditions at later stages, implying a role in basal developmental regulation rather than direct auxin responsiveness (**Figure 10B**). Notably, the magnitude of expression changes in stems was substantially greater than in leaves, with some genes exhibiting more than 10-fold induction, highlighting enhanced auxin sensitivity in stem tissues.

## 4 Discussion

Growth hormones have an important role in influencing plant development from embryonic stage to senescence. Auxin is one of the hormones, essential for plant growth and development, modulating responses to environmental stimuli and guiding organ growth through the establishment and maintenance of auxin concentrations. Auxin response factors (ARF) are transcriptional factors that control the expression of auxin responsive genes (Hu et al., 2025). Recently in many plant species *ARF* genes are reported and their members are varied from species to species. For instance, 24, 25, 81, and 216 *ARFs* genes are reported in *Canna generalis, O. sativa, Medicago sativa*, and *Bambusa*, respectively (Wang et al., 2026; Chen F. et al., 2023; Hu et al., 2025; Wang et al., 2007). The present study provides a comprehensive genome wide characterization of the *ARF* gene family in *S. officinarum* LA-Purple, offering new insights into the evolutionary expansion and regulatory complexity of auxin. Signaling components in a high polyploid crop. The identification of 73 *SoARF* genes represents a substantial expansion compared to the diploid species, reinforcing the hypothesis that polyploidization has played a central role in shaping TF repertoires in sugarcane. The considerable variation in SoARF protein length, ranging from 661 to 1155 amino acids, may reflect structural diversification among family members rather than random variation alone. Because all retained SoARF proteins contained conserved ARF-related domains, including the B3 DNA-binding domain, Auxin_resp domain, and AUX/IAA-related region, the length differences are likely associated with variation in non-conserved regions, motif organization, and exon–intron structure. Such variation may contribute to functional divergence among duplicated *SoARF* genes, particularly in transcriptional activation or repression capacity and protein–protein interaction potential. Nevertheless, given the highly polyploid and complex nature of the sugarcane genome, possible annotation uncertainties cannot be completely excluded, and functional validation will be required to confirm the biological significance of these structural differences. This expansion is consistent with regulatory capacity and functional redundancy, potentially buffering developmental processes under fluctuating environmental conditions (Vicient and Casacuberta, 2017).

Gene duplication is an evolutionary process that creates extra copies of genes, enabling them to specialize in already existing functions or evolve new ones. This method permits mutations without having an adverse effect right away while maintaining vital biological functions (Copley, 2020). By altering gene expression patterns, regulatory divergence increases genomic complexity, functional innovation, and adaptive potential, which in turn promotes the formation of new biological pathways and characteristics. Duplication events at various sizes including single-gene, segmental, and whole-genome duplications have significantly influenced the genomic architecture and genes content of the plants (Van de Peer et al., 2021; Xia et al., 2025). The predominance of segmental duplication and inter-chromosomal synteny over tandem duplication suggests that whole-genome duplication (WGD) events have been the primary evolutionary force driving *ARF* family expansion in *S. officinarum* LA-Purple. This finding aligns with recent genomic studies in sugarcane and related grasses, where polyploidy is associated with extensive gene retention and sub-functionalization (Chen H. et al., 2023; Ding et al., 2024; Zhai et al., 2023). Notably, the presence of one-to-many orthologous relationships with *S. spontaneum* and *S. bicolor* further supports asymmetric gene retention following duplication, which is a characteristic of polyploid genome evolution. However, the absence of syntenic counterparts for certain *SoARF* genes raises the possibilities of lineage-specific innovations or gene loss, suggesting ongoing dynamic evolution within the ARF family. Phylogenetic analysis revealed that *SoARF* genes are predominantly clustered into groups II and IV with complete absence in the groups I and III. The dominance of group IV members may indicate functional specialization or expansion of specific auxin-responsive pathways in sugarcane. Moreover, the clustering of *SoARFs* with homologs from *S. spontaneum*, *S. bicolor*, and *Z. mays* confirms strong evolutionary conservation across monocots, while also highlighting divergence within sugarcane species. Similar phylogenetic classification also reported in sugarcane (Lin et al., 2023).

Structurally, the conservation of key domains such as B3 DNA binding domain and AUX/IAA interaction domain across most SoARF proteins confirm their canonical role in the auxin signaling (Rienstra et al., 2025). However, variation in the motif composition and exon-intron organization suggest functional diversification among the family members. This suggest that duplicated genes may have undergone sub-functionalization or neo-functionalization. The presence of additional domain (PRK10263) in some members suggest that the protein belongs to a conserved TF family, likely associated with auxin response pathways and acquired novel regulatory roles. However, functional validation is required to determine whether these structural variations translate into distinct translational activities. Functions of genes depends on *Cis*-regulatory elements in the promoter regions (Kaur and Pati, 2016). Our findings of cis-regulatory elements revealed. A complex and multilayered regulatory system including hormone-responsive, stress related and developmental elements across the *SoARF* promoters. Many *cis*-elements were involved in the hormone’s responses, such as TCA-element, ABRE, AuxRE. Additionally, different stress related elements such as MBS in drought (Tang et al., 2023), DRE responsive to dehydration (Han et al., 2022), and LTR in low temperature (Wu et al., 2019) were reported. This suggests that *SoARFs* may act as integrative hubs linking hormonal signaling with environmental stress responses. This is consistent with emerging evidence that *ARFs* participate in cross-talk between auxin and other signaling pathways including abscisic acid and salicylic acid pathways (Mazzoni-Putman et al., 2021; Ding et al., 2025). Although stress-responsive cis-elements were identified in *SoARF* promoters, no abiotic stress-expression data were included in this study. Therefore, their stress-related roles remain predictive and require validation under drought, salinity, and other stress conditions.

Small non-coding RNAs known as microRNA (miRNA) use transcript cleavage or transcriptional repression to control target gene expression at the transcriptional stage (Alaei and Kakumani, 2025). The prediction of miRNA-*SoARF* interaction network highlights an additional layer of post-transcriptional regulation. In the current study, the prominent targeting of *SoARFs* by miRNA160 and miRNA167 families is consistent with conserved regulatory modules reported in multiple plant species, underscoring their central role in fine-tuning auxin responses. In a previous study of potato, it was reported that the miRNA160 regulates the antagonistic crosstalk between auxin mediated growth and salicylic acid mediated defensive responses and is essential for late blight pathogen (Natarajan et al., 2018). Similarly, miRNA167 was involved in seed development (Yao et al., 2019). Other families including miRNA169, miRNA171, miRNA396, and miRNA444 were also found targeting specific *SoARFs*, which were reported in stress tolerance (Samynathan et al., 2023). Notably, the *SoARF11*, *SoARF14*, and *SoARF16*, were targeted by 25 different types of miRNAs. PPI analysis further supports the integration of *SoARF* into complex regulatory networks. The identification of central hub proteins interacting with multiple *SoARFs* suggest coordinated regulation of downstream transcriptional responses. Nevertheless, the reliance on ortholog-based inference from *S. bicolor* limits the specificity of these predictions for sugarcane, and experimental validation is necessary to confirm these interactions *in vivo*.

Expression profiling provides functional insight into *ARF* gene roles in development and auxin signaling. Recent studies have proved that *ARF* genes have regulatory roles in abiotic stresses. The overexpression of *EgARF9, EgARF10, EgARF12, EgARF13, EgARF15*, and *EgARF22* in palm increases the drought and salt tolerance (Jin et al., 2022). Similarly, in *Cucubita pepo* L. 94% and 88% *CpARFs* genes were up regulated under salt and drought stresses, respectively (Zhang et al., 2024). In this study, the differential expression of *SoARF* genes across tissues and developmental stages indicates strong spatial and temporal regulations. Genes such as *SoARF1, SoARF2, SoARF3*, and *SoARF7* consistently showed high expression, suggesting core role in growth and development. In contrast, low or non-responsive genes may represent either condition specific regulators or pseudogenization in a polyploid context. Under the exogenous IAA treatment, the dynamic expression patterns of several *SoARFs* confirm their responsiveness to auxin signaling. The presence of both induced and repressed genes highlights the dual role of *ARFs* as transcriptional activators and repressors. Temporal variations in the expression pattern indicates a finely tuned regulatory system, where different *SoARF* may function at distinct stages of the auxin response.

In addition, RNA-seq analysis of *S. officinarum* LA-Purple demonstrated that numerous *SoARF* genes respond to exogenous IAA treatment, indicating their involvement in auxin signaling pathways. To further validate these expression trends, qRT-PCR analysis was conducted using the widely cultivated sugarcane cultivar GT58. Although different genetic backgrounds were used, this approach was intentional because modern sugarcane cultivars possess highly complex polyploid and aneuploid genomes (2n = 100–130), with approximately 80% of their genome inherited from *S. officinarum* and 10–15% from *S. spontaneum* (Zhang et al., 2018). Consequently, absolute transcript levels may vary among genotypes, whereas conserved expression responses across cultivars provide stronger evidence for the biological relevance and robustness of candidate genes. The qRT-PCR results largely supported the RNA-seq observations and confirmed that auxin regulation within the *SoARF* family is highly selective rather than uniform. Several genes including *SoARF2*, *SoARF*6, *SoARF12*, and *SoARF19* showed increased transcript accumulation under IAA treatment, indicating these members may function as positive auxin responsive regulators. The stronger induction at later time points, particularly 48 h and 96 h, further suggests a time dependent activation pattern, possibly reflecting secondary transcriptional responses after initial auxin perception. In contrast, *SoARF43*, *SoARF44*, *SoARF48*, and *SoARF71* were consistently repressed by IAA treatment, indicating that they may function as negative regulators within auxin signaling pathways. The spatiotemporal expression patterns of *SoARF* genes provide important insights into their potential roles during sugarcane development, particularly because sugarcane is a vegetatively propagated crop characterized by rapid stem elongation, continuous biomass accumulation, and distinct developmental gradients along the stalk. In the present study, several *SoARF* genes, including *SoARF1*, *SoARF2*, *SoARF3*, *SoARF5*, *SoARF6*, and *SoARF7*, showed high expression in mature leaf and developing stem tissues, suggesting their involvement in auxin-mediated regulation of vegetative growth and tissue differentiation. This expression pattern is consistent with the established role of auxin in leaf expansion, vascular development, internode elongation, and stem growth. Conversely, genes preferentially expressed in mature stem tissues may contribute to stalk maturation and biomass accumulation. These findings also align with recent sugarcane functional genomics approaches that use subgenome-aware transcriptomics and co-expression analysis to link *Saccharum*-derived loci with agronomic traits. Therefore, the IAA-induced *SoARF1*, *SoARF2*, *SoARF6*, *SoARF7*, *SoARF12*, and *SoARF19*, together with auxin-repressed *SoARF43*, *SoARF44*, *SoARF48*, and *SoARF71*, should be prioritized for allele-specific expression analysis, promoter haplotype analysis, and functional validation in sugarcane backgrounds (Hui et al., 2024). Overall, the tissue- and stage-specific expression patterns indicate that functional diversification of *SoARF* genes may facilitate developmental regulation across different growth stages of sugarcane and provide promising candidates for improving agronomically important traits, including stalk growth, biomass production, and plant architecture. Importantly, the integration of promoter composition, miRNA targeting, PPI prediction, and expression profiling suggests that the *SoARF* family is not merely expanded at the genomic level but may also be functionally partitioned across developmental contexts. The high expression of *SoARF1*, *SoARF2*, *SoARF3*, *SoARF6*, *SoARF7*, *SoARF12*, and *SoARF19* in specific tissues or under IAA treatment supports their potential involvement in auxin-mediated developmental processes, particularly leaf development and stem growth. In contrast, genes showing weak or repressed expression under IAA treatment may represent negative regulators, redundant paralogs, or members with functions restricted to other developmental or stress conditions. Thus, the combined evidence provides a candidate-gene framework for future functional studies rather than only a descriptive catalog of the *SoARF* family.

## 5 Conclusions

In this study, 73 *ARF* genes in *S. officinarum* LA-Purple were identified using a genome-wide analysis, which showed a significant expansion in *ARF* gene family in domesticated sugarcane. In-depth characterization revealed that these *SoARF* genes maintained the conserved *ARF*-associated domains, but had a high degree of gene structure, protein properties, and promoter architecture variation, showing evolutionary conservation and functional branching. The promoter analysis revealed that there are 49 *cis*-regulatory elements related to hormone responsiveness, development, and signaling due to stress conditions, indicating that *SoARFs* might act as crucial regulatory nodes that connect auxin signaling to environmental and developmental responses. In addition, widespread miRNA targeting, especially of miR160 and miR167 families, and predicted PPI networks, indicated the multilayered regulatory processes involved in the functions of *SoARF*. Further expression profiling with exogenous IAA treatment, and among various tissues and developmental stages demonstrated unique spatial and temporal patterns of expression, indicating conserved and specialized functions among *SoARF* members. Interestingly, *SoARF1*, *SoARF2*, *SoARF3*, *SoARF6, SoARF7*, *SoARF12*, and *SoARF19* genes have been identified as promising regulators of auxin responsiveness and developmental regulation. Additionally, confirmation by qRT-PCR under IAA treatment indicated that some *SoARF* genes were differentially expressed in response to auxin, with greater transcriptional changes observed in stems than in leaves. Collectively, our results support the hypothesis that expansion of the *SoARF* gene family through polyploidization was accompanied by regulatory diversification and differential expression among developmental tissues. The identification of aforementioned highly expressed and auxin-responsive candidates, suggests that these genes may play important roles in auxin-mediated developmental regulation in sugarcane. This comprehensive characterization of the *ARF* gene family in *S. officinarum* LA-Purple establishes a valuable foundation of functional validation, targeted genetic improvement, and molecular enhancement of sugarcane in the future.

## Data Availability

The original contributions presented in the study are included in the article/**Supplementary Material**. Further inquiries can be directed to the corresponding author/s.
