# Supplementary material for: Integrative analysis of SoARF gene family uncovers their role in hormone signaling and development in sugarcane
Source: Front Plant Sci. 2026 Jun 26;17:1874213. doi: 10.3389/fpls.2026.1874213 (PMC13350516; doi:10.3389/fpls.2026.1874213)
Supplement: Supplementary file 1 [file Presentation1.pptx]

## Slide 1
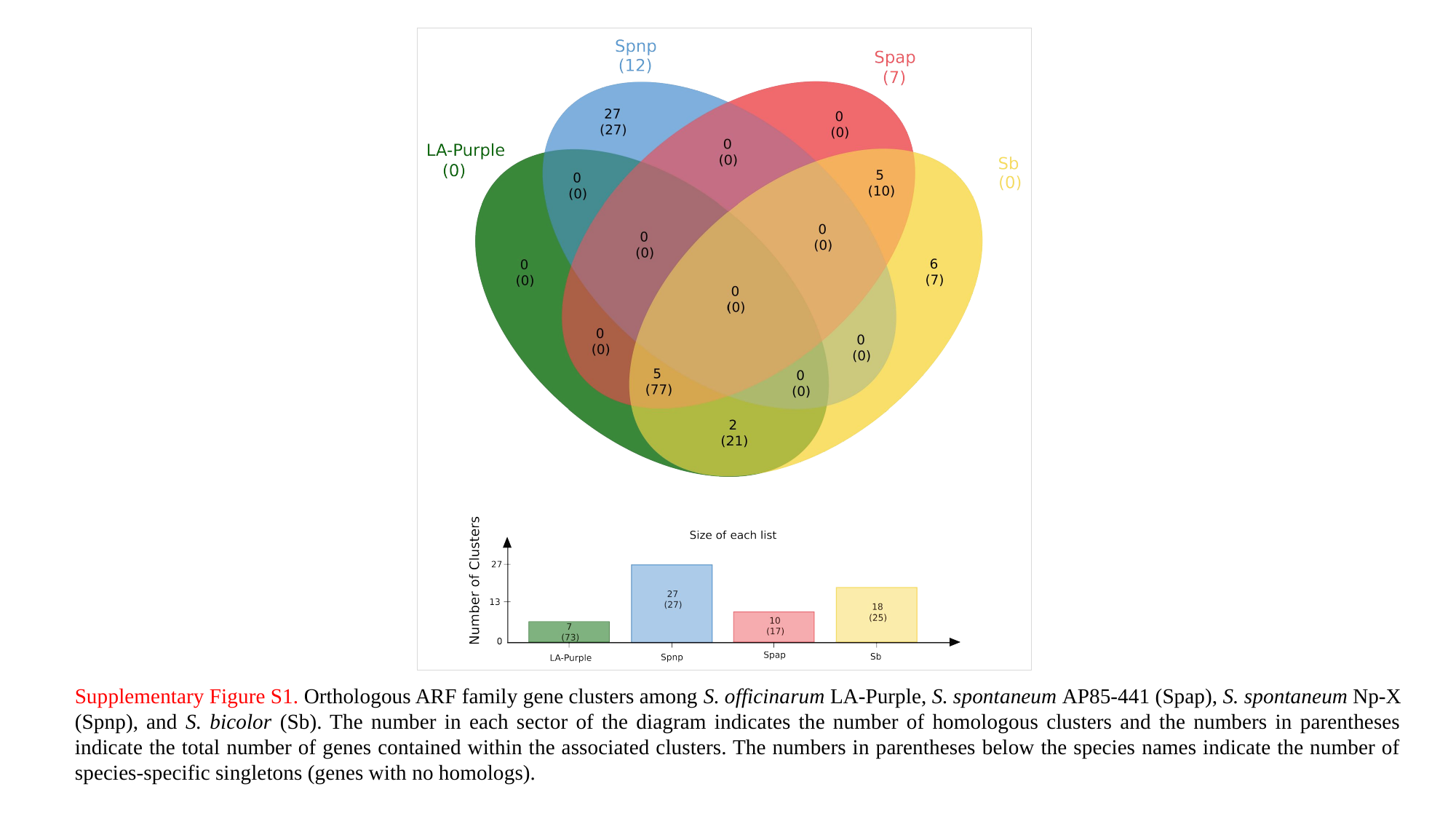

Supplementary Figure S1. Orthologous ARF family gene clusters among S. officinarum LA-Purple, S. spontaneum AP85-441 (Spap), S. spontaneum Np-X (Spnp), and S. bicolor (Sb). The number in each sector of the diagram indicates the number of homologous clusters and the numbers in parentheses indicate the total number of genes contained within the associated clusters. The numbers in parentheses below the species names indicate the number of species-specific singletons (genes with no homologs).
