# Supplementary material for: Integrative analysis of SoARF gene family uncovers their role in hormone signaling and development in sugarcane
Source: Front Plant Sci. 2026 Jun 26;17:1874213. doi: 10.3389/fpls.2026.1874213 (PMC13350516; doi:10.3389/fpls.2026.1874213)
Supplement: Supplementary file 2 [file Presentation2.pptx]

## Slide 1
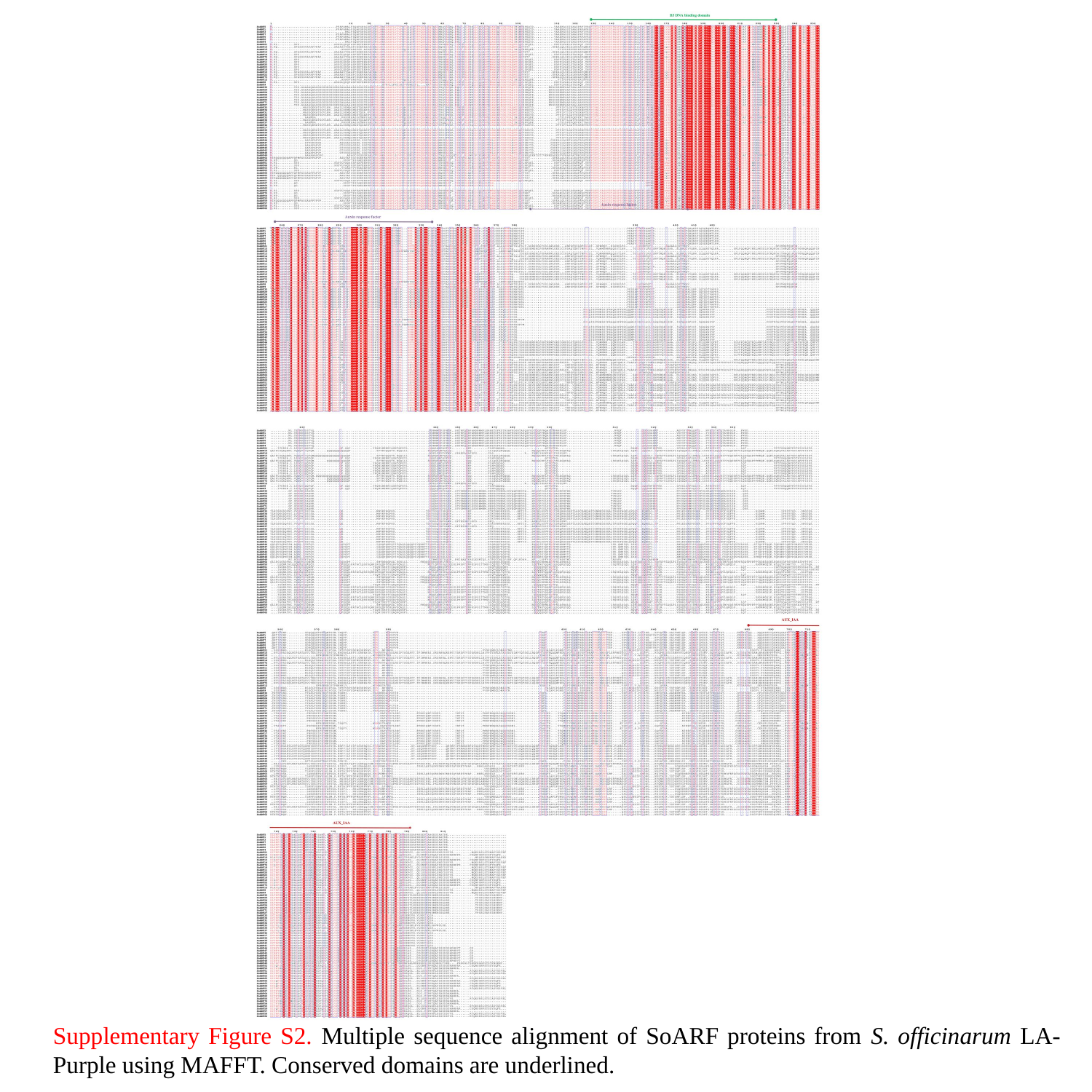

Supplementary Figure S2. Multiple sequence alignment of SoARF proteins from S. officinarum LA-Purple using MAFFT. Conserved domains are underlined.
