# Supplementary material for: Integrative analysis of SoARF gene family uncovers their role in hormone signaling and development in sugarcane
Source: Front Plant Sci. 2026 Jun 26;17:1874213. doi: 10.3389/fpls.2026.1874213 (PMC13350516; doi:10.3389/fpls.2026.1874213)
Supplement: Supplementary file 3 [file Presentation3.pptx]

## Slide 1
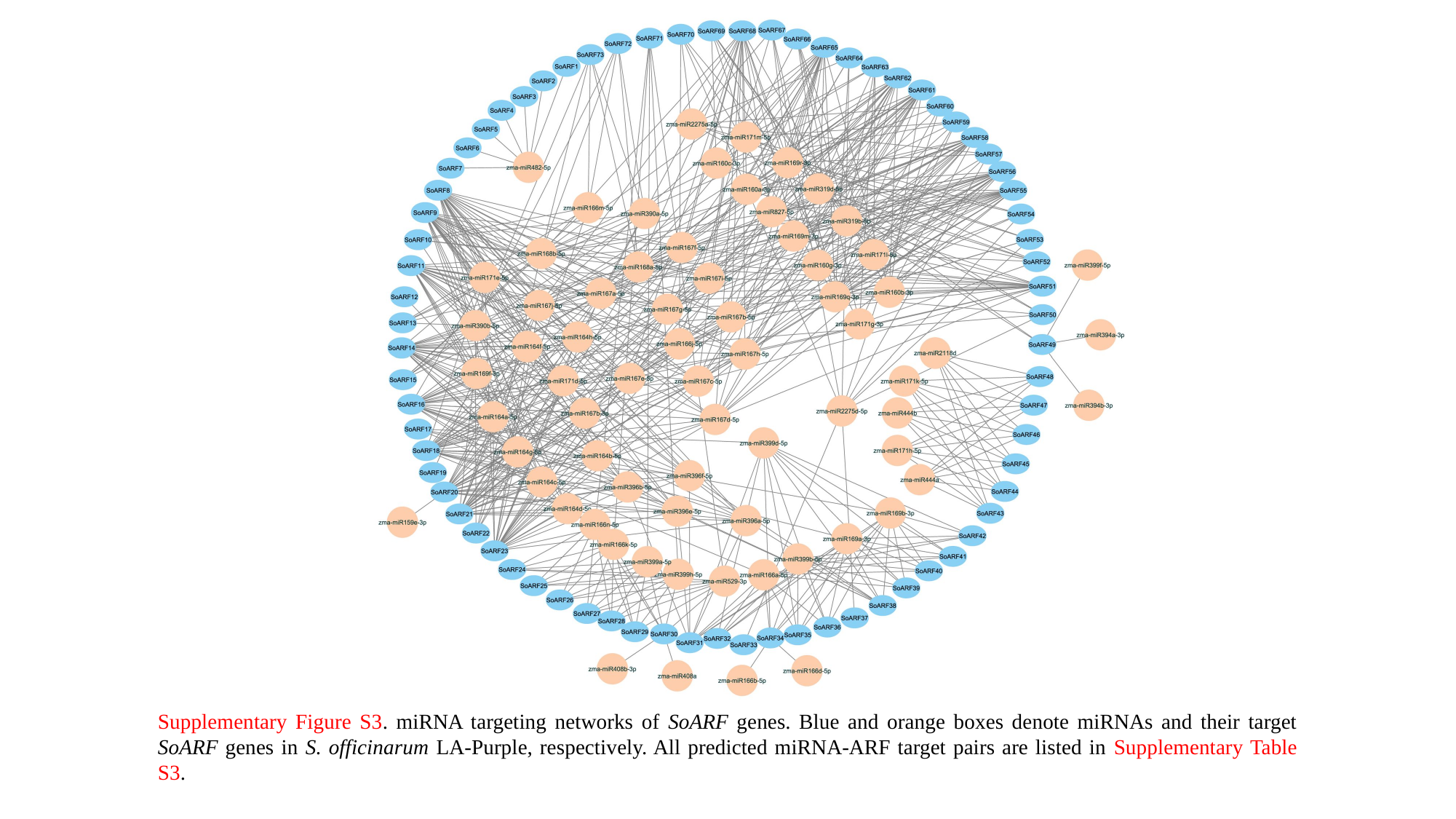

Supplementary Figure S3. miRNA targeting networks of SoARF genes. Blue and orange boxes denote miRNAs and their target SoARF genes in S. officinarum LA-Purple, respectively. All predicted miRNA-ARF target pairs are listed in Supplementary Table S3.
